# Genome mining the black-yeast *Aureobasidium pullulans* NRRL 62031 for biotechnological traits

**DOI:** 10.1186/s12864-025-11395-2

**Published:** 2025-03-13

**Authors:** Difan Xiao, Marielle Driller, Karla Stein, Lars M. Blank, Till Tiso

**Affiliations:** iAMB - Institute of Applied Microbiology, ABBt - Aachen Biology and Biotechnology, RWTH Aachen University, Worringer Weg 1, Aachen, 52074 Germany

**Keywords:** *Aureobasidium pullulans*, Whole-genome sequence, Phylogeny, Functional annotation, Biosynthetic gene clusters

## Abstract

*Aureobasidium pullulans* is a yeast-like fungus known for its commercial biomanufacturing of pullulan. This study explores the genome of *A. pullulans* NRRL 62031, highlighting its biosynthetic potential, metabolic pathways, and physiological traits. Additionally, it demonstrates actual product formation and links molecular features to biotechnological applications. Phylogenetic analysis suggested it might be closely related to *Aureobasidium melanogenum*. While the functional annotation revealed a wide carbohydrate catabolism, growth evaluation demonstrated that the microbe can utilize not only saccharides but also polyols and organic acids. The extracellular cellulolytic, xylanolytic, and pectinolytic activities were indicated by the formation of visible halos on agar plates. The antiSMASH pipeline, NCBI Blastp alignment, and product qualification confirmed that *A. pullulans* NRRL 62031 can produce melanin, pullulan, polymalate, and polyol lipids. Moreover, yanuthone D, burnettramic acid A, choline, fructooligosaccharides, gluconic acid, and β-glucan might be synthesized by *A. pullulans* NRRL 62031. The results clearly show the extraordinary potential of *A. pullulans* NRRL 62031 as a microbial chassis for valorizing biomass residues into value-added bioproducts. The strong catabolic and anabolic capacities indicate significant promise for biotechnological applications. The results are discussed in the context of metabolic engineering of *Aureobasidium*.

**Supplementary Information:**

The online version contains supplementary material available at 10.1186/s12864-025-11395-2.

## Introduction

Nowadays, microbial biotechnology is employed as an important alternative to petroleum-based chemical processes [1]. However, achieving high yields, a low CO_2_ footprint, and ultimately low cost of goods sold (COGS) often remains a challenge in biotechnology. Simple fermentations on inexpensive carbon sources are required to contribute to the envisaged bioeconomy [2]. The saprophytic yeast-like *Aureobasidium pullulans* has been suggested as a potential fungal chassis for biotechnology due to its highly versatile metabolic network [3–5]. Strains of *A. pullulans* are widely distributed in the phyllosphere (*e.g.*, leaves and wood), in the lithosphere (*e.g.*, limestone, rocks, and even monuments), and in aquatic environments (*e.g.*, coastal waters and fresh waters) [3, 6, 7]. Moreover, they have also been isolated from extreme surroundings like hypersaline habitats, the deep sea, and glacial ice [8–11]. The genus *Aureobasidium* is a member of the family Aureobasidiaceae, the order Dothideales, and the phylum Ascomycota, which comprises 27 taxa (species and varieties) [4, 12].

In the past, according to differences in morphology, physiology, and metabolite production, the genus *Aureobasidium* was taxonomically divided into three species: *Aureobasidium pullulans*, *Aureobasidium proteae*, and *Aureobasidium leucospermi*. *A. pullulans* has four varieties, namely, *A. pullulans* var. *pullulans* [13], *A. pullulans* var. *melanogenum* [14], *A. pullulans* var. *subglaciale* [11], and *A. pullulans* var. *namibiae* [11]. However, Gostinčar et al. [4] redefined these varieties as separate species: *A. pullulans*, *A. melanogenum*, *A. subglaciale*, and *A. namibiae* based on a comparison of their whole-genome sequences. It is to be expected that more species and varieties will be discovered in the future as many strains of *Aureobasidium* spp. have been identified recently, including *Aureobasidium mangrovei* [15], *Aureobasidium khasianum* [16],* Aureobasidium hainanensis* [17], and* Aureobasidium thailandense* [18]. *A. pullulans* is the most abundant, well-studied, and ubiquitous species within the genus [19], generally displaying three types of cell morphology: elliptical yeast-like cells, branched septate filaments, and thick-walled chlamydospores [3]. The colonies of *A. pullulans* initially are observed as light pink, cream-coloured, or light brown, followed by becoming blackish owing to melanin production at a later growth stage. The formation of heavily melanized chlamydospores is a prominent characteristic of the genus [3, 20, 21]. Currently, *A. pullulans* has been known for the commercial production of its trademark product, pullulan, a biodegradable extracellular homopolysaccharide consisting of maltotriose repeating units interconnected by α-(1 → 6) linkages [22–24]. Pullulan is widely used in food, agriculture, pharmaceuticals, medicine, and cosmetics due to its structural flexibility, edibility, biodegradability, and oxygen barrier properties [25–27]. Other less exploited secondary metabolites from different strains of *A. pullulans* include polymalate [28–30], the biosurfactant polyol lipids, a.k.a. liamocins [31–36], and melanin [37, 38].

Polymalate (PMA), a promising second-generation biomaterial, is a linear biodegradable polyester formed by the interlinkage of L-malate units, which can be employed in surgical sutures, novel drug carriers, biodegradable plastics, coating materials, and other advanced biomaterials [39–41]. Polyol lipids are composed of a single polyol headgroup (*e.g.*, mannitol or arabitol) linked to either three or four 3,5-dihydroxydecanoic ester groups. These glycolipids have potential as biosurfactants and they have been reported to exhibit antimicrobial and anticancer activities [32, 42, 43]. The melanin synthesized in *A. pullulans* is mainly dihydroxynaphthalene (DHN)-melanin, an insoluble heterogeneous biopolymer without a defined molecular structure [44]. It has significant physiological and biological functions. For instance, it has antitumor, antiradiation, antiviral, and antioxidant activities and facilitates tolerance to UV radiation, high temperatures, high salt concentrations, oxidative agents, drought, and antibiotics, which is one of the reasons that some strains of *A. pullulans* are able to survive in diverse hostile surroundings [45, 46]. Moreover, melanin might be utilized as a potent antioxidant agent substituting astaxanthin and other antioxidants [47]. From the perspective of biodegradation, various strains of *A. pullulans* isolated from different habitats have been confirmed to possess an extensive repertoire of hydrolytic enzymes for the hydrolysis of abundant and cheap plant biomass, including cellulose, xylan, starch, inulin, and pectin [47]. Reported CAZymes (carbohydrate-active enzymes) include extracellularly secreted cellulases [48–50], xylanases [51–53], amylases [54, 55], lipases [3, 56, 57], laccases [58], mannanases [3], and proteases [59–61]. The ongoing search for new strains of *A. pullulans* might lead to the discovery of other valuable metabolites and extracellular enzymes of industrial interest.

Over the past decades, several whole-genome sequencing projects on *A. pullulans* have been undertaken. The first draft genome sequence of *A. pullulans* was annotated for strain AY4 in 2012, containing around 26.7 Mbp, with an average GC content of 50% [62]. In 2014, de novo genome sequencing of four varieties of *A. pullulans* was performed. The genome data were used for taxonomic placement, biotechnological potential assessment, and stress tolerance analysis [4]. Gostinčar et al. analyzed the genome sequence data of fifty *A. pullulans* strains from different habitats to substantiate that *A. pullulans* are generalistic fungi that can adapt to diverse habitats without significant intraspecific specialization [63]. In the same year, gluconic acid-producing strain *A. pullulans* P25 was whole-genome sequenced and annotated. It was found to be phylogenetically close to *A. pullulans* EXF-150 [64]*.* Of late, the whole-genome and mitochondrial sequences of *A. pullulans* var. *aubasidani* CBS 100524 were acquired. This variety can secrete aubasidan [65]. Recently, the resource of the whole genome of *A. pullulans* NRRL 62031 was announced [66].

We performed bioinformatic data mining for *A. pullulans* NRRL 62031 to predict its biosynthetic and biodegradable abilities and metabolic versatility on different carbon sources. These predictions were further substantiated by wet-lab experiments. Functional gene prediction was carried out for the following two categories: plant biomass depolymerization including cellulose, xylan, starch, and pectin, and biosynthesis for melanin, pullulan, polymalate, and polyol lipids. Furthermore, the respective capabilities of hydrolysis and biosynthesis were experimentally confirmed. Some biosynthetic gene clusters and key enzyme-encoding genes relevant to other metabolite synthesis including two antibiotics, choline, fructooligosaccharides, gluconic acid, and β-glucan were also annotated. In addition, we conducted a comparative genomic analysis between *A. pullulans* NRRL 62031 and other species of *Aureobasidium* spp. to understand its phylogenetic position and evolutionary status. The results of this study suggest that *A. pullulans* NRRL 62031 has the potential to serve as a versatile microbial cell factory for converting cheap and easily available feedstocks into a wide array of useful compounds. In silico data obtained from the whole-genome sequence also provide insights into possible future enhancements regarding the synthesis of value-added commodity chemicals and substrate utilization through metabolic engineering and synthetic biology.

While other studies have already analyzed genomes of different *Aureobasidium* strains [4, 62, 63], we here use an approach, which offers greater accuracy and is more systematic in defining the taxonomic status [4]. Additionally, this study integrates more public databases for gene function prediction.

## Materials and methods

### Microorganism, growth conditions, and media

*A. pullulans* NRRL 62031 was purchased from the Agricultural Research Service Culture Collection (Peoria, Illinois, USA). It was originally obtained from a leaf in Nakornratchasima, Thailand by Pennapa Manitchotpisit in January 2010 [40]. The medium for seed culture was a YPD medium containing 20.0 g/L glucose, 20.0 g/L peptone, and 10.0 g/L yeast extract. The recipe of media for the production of polymalate, pullulan, and melanin was referred to corresponding literature [67–69]. The medium for the cell growth assay was minimal medium (2.0 g/L of NH_4_NO_3_, 0.1 g/L of KH_2_PO_4_, 0.1 g/L of MgSO_4_·7H_2_O, 0.5 g/L of KCl, and 20.0 g/L of carbon source), which was adapted from the PMA production medium [67]. The extra 20.0 g/L agar powder was supplemented to prepare solidified plates. All Erlenmeyer flask cultivations were carried out under aerobic conditions on a Multitron shaker (INFORS, Bottmingen, Switzerland) at 30°C with a 200 rpm shaking speed. The chemicals used were purchased from Carl Roth (Karlsruhe, Germany), Sigma-Aldrich (St. Louis, Missouri, United States), or Merck (Darmstadt, Germany) unless stated otherwise.

### Genomic DNA preparation and de novo genome sequencing

Prior to genome sequencing, a single fresh colony was inoculated in yeast extract-peptone-dextrose (YEPD) broth medium at 30°C overnight. The genomic DNA (gDNA) was extracted from freshly harvested cell pellets with the PureLink Genomic DNA Kit (Invitrogen, CA, USA) following the manufacturer’s guidelines. The mild acoustic shearing was employed for gDNA fragmentation with a Covaris S220 instrument (Covaris, Inc., Woburn, MA, USA). The resulting fragmented DNA was cleaned up and end-repaired using an Agilent 2100 Bioanalyzer (Agilent Technologies, Palo Alto, CA, USA). Adapters were ligated after adenylation of the 3’ends followed by enrichment by limited cycle PCR. DNA libraries were validated using a High Sensitivity D1000 ScreenTape on the Agilent TapeStation (Agilent Technologies, Palo Alto, CA, USA) and were quantified using Qubit 2.0 Fluorometer (Thermo Fisher Scientific, Carlsbad, CA, USA) as well as real-time PCR (Applied Biosystems, Carlsbad, CA, USA). The libraries passing the quality control were subsequently clustered and loaded on the Illumina HiSeq instrument. The qualified libraries were sequenced using a 2 × 150 paired-end configuration. Image analysis and base calling were conducted using the HiSeq Control Software (HCS). The raw sequence data (.bcl files) generated from Illumina HiSeq was converted into standard FASTQ files and de-multiplexed using bcl2fastq 2.17 software.

### Genome assembly, gene prediction, annotation, and DNA repeat element analysis

The adapter sequences and low-quality sequences were removed to obtain high-quality clean data using cutadapt (v1.9.1). Depending on the clean data, the k-mer analysis was performed using Velvet (v1.2.10), and the de Brujin plot was constructed using the overlapping relationship between the k-mers. The assembled genome was in the format of fragmented contigs. SSPACE (v3.0) was used to align the sequencing reads to the contigs, which were subsequently assembled into scaffolds depending on the pairwise relationship between the paired-end reads and the size of the inserted segments. GapFiller (v1.10) was utilized to align all the reads from the library to the scaffold sequences. The alignment was used to fill the gaps in the scaffolds and extend the scaffold sequences to acquire longer ones with a lower rate of undetermined (N) bases. The whole-genome shotgun project has been deposited at DDBJ/ENA/GenBank under the accession no. JALBUZ000000000. The version described in this paper is version no. JALBUZ010000000. The respective raw sequencing data have been deposited at the Sequence Read Archive under the accession number no. SRR17771678.

The finally optimized assembly data of *A. pullulans* NRRL 62031 was subjected to the prediction of protein-encoding genes, tRNAs, rRNAs, and other non-coding RNAs using Augustus (v3.3), tRNAscan-SE (v1.3.1), Barrnap (v0.9), and Rfam (v12.2), respectively. The BLAST (v2.2.31) [70] was performed with an E-value less than 1*e^−5^ against the Kyoto encyclopedia of genes and genomes (KEGG) for metabolic pathways [71], gene ontology (GO) for annotation of the homologous genes and their function, location of cellular components and biological processes [72], KOG (eucaryotic orthologous groups) for eukaryotic clusters of orthologues [73], NCBI-NR (non-redundant protein database) for protein alignments [74], Swiss-Prot for mapping the gene-ontology terms [75], CAZymes (carbohydrate-active enzymes) for describing structurally-related catalytic and carbohydrate-binding modules (or functional domains) of enzymes [76], Pfam (protein families database) for classifying protein sequences into families and domains [77], ATFDB (*Aureobasidium* transcription factor database) for identifying transcription factors [78], and TCDB (transporter classification database) for predicting membrane transport proteins.

The DNA repetitive sequences in the genome were predicted from scratch using RepeatModeler (version 1.0.8) [79]. The analysis process proceeded with two steps. The first step was to identify potential repeat elements using RECON [80] and RepeatScout (version 1.0.5) [81] and to construct a library based on the optimized preliminary result using RepeatModeler. The second step was to search and analyze the DNA elements in the target genome using RepeatMasker (version 4.0.5) (http://www.repeatmasker.org). Meanwhile, NUCMER (Nucleotide Mummer) (version 3.1) [82] was employed for further screening of the repeats to remove the sequences over 100bp long.

### antiSMASH analysis

The current version of antiSMASH 7.1.0 (Antibiotics and Secondary Metabolites Analysis SHell) allows the rapid identification, annotation, and analysis of secondary metabolite biosynthesis gene clusters (BGCs) at a genome-wide level [83, 84]. The final assembly whole-genome data in FASTA format was uploaded to the antiSMASH 7.1.0 program (https://fungismash.secondarymetabolites.org/) with default parameters and relaxed detection strictness.

### Phylogeny and comparative genome analysis

For phylogenetic analysis, the core genes (genes that are present in all genomes analyzed) of *A. pullulans* NRRL 62031 and 16 publicly available genome sequences of *Aureobasidium* spp. were determined (Table S1) and a phylogenetic tree was computed based on 5,415 core genes using the comparative genomics platform EDGAR 3.2 [85, 86]. Within the EDGAR workflow, the core genes are determined by first selecting the genome of *A. pullulans* NRRL 62031 as the reference. Then, all CDS from this reference genome undergo a reciprocal best blast hit analysis against another selected genome. Only the CDS with reciprocal best blast hits are retained. This process iterates with all genomes to be considered for core gene identification until all genomes have been compared to the remaining set of CDS. For the phylogenetic tree, alignments of each core gene set using MUSCLE were generated followed by a combination of the alignments to one huge alignment. With this alignment as input, an approximately-maximum-likelihood tree was constructed using the FastTree software [87]. Local support values were computed using the Shimodaira-Hasegawa (SH) test. In addition to the phylogenetic tree, EDGAR 3.2 was also employed to perform an Average Nucleotide Identity (ANI) analysis. The calculation of the ANI matrix is based on a BLASTN comparison of the genome sequences as described by Goris et al*.* [88], facilitating the determination of relationships between different species [89].

### Cell growth measurements

The precultures of *A. pullulans* NRRL 62031 were inoculated in 24-well plates filled with 2 ml of minimal medium added with 2% (w/v) carbon sources, followed by loading in the Growth Profiler 960 (Enzyscreen, Heemstede, The Netherlands) run at 30°C, 225 rpm shaking speed. The initial OD_600_ was set to 0.2. The software Growth Profiler Control v4_9_0 was used to analyze the online growth of yeast-like cells. The Growth Profiler was set to generate a scan of the plate every 30 min. Based on this scan, the density of cells was expressed as green value (G-value), which was calculated from imaging analysis of microtiter plates with transparent bottoms. A calibration curve was generated in order to convert the G-values into OD_600_ values. The maximal growth rates (µ) were determined by fitting an exponential curve to a plot of OD_600_ over time of cultures in the exponential phase. The experiment was carried out in duplicate.

### Detection of extracellular enzyme activities

The halo visualization method using Gram’s iodine was employed to detect extracellular cellulolytic, xylanolytic, and pectinolytic activities [90]. Gram’s iodine formed a bluish-black complex with unhydrolyzed raw materials but not with the hydrolyzed part of polysaccharides, giving a sharp and distinct zone around the microbial colonies within 3 to 5 min. Briefly, 10 µl overnight grown precultures were spotted on solid plates (0.2% NaNO_3_, 0.1% K_2_HPO_4_, 0.05% MgSO_4_, 0.05% KCl, 0.02% peptone, and 1.7% agar) supplemented with 2% (w/v) CMC (carboxymethyl cellulose), Avicel (microcrystalline cellulose), beechwood xylan, and pectin from citrus peel. Plates were incubated for 24 h at 30°C, followed by flooding with Gram’s iodine for 5–10 min. *Saccharomyces cerevisiae* BY4743 and *Escherichia coli* DH5α were used as a negative controls. The experiment was performed in triplicate.

### Purification and enzymatical hydrolysis of pullulan

The culture after 7 days obtained from the EPS production medium (50 ml) was centrifuged at 10,000 × g and 4°C for 10 min to remove cells and other precipitates. 10 ml supernatant was mixed with 20 ml 95% (v/v) cold ethanol and kept at 4°C for 24 h to precipitate the putative pullulan. After removal of the ethanol, the resulting precipitate was dissolved in 10 ml deionized water at 90°C and followed by 20 ml 95% (v/v) cold ethanol precipitation. The above procedures were repeated three times. The final purified putative pullulan was desiccated using a vacuum pump [91]. The purified precipitate (0.1 g) was thoroughly dissolved in 10 ml of deionized water at 90°C. The dissolved substrate was hydrolyzed by incubating the mixture of 1.0 ml of the substrate, 0.9 ml of 50 mM sodium acetate and acetic acid buffer (pH 4.5), and 0.1 ml of pullulanase (Sigma-Aldrich, St. Louis, Missouri, United States) for 15 min at 60°C [92]. The standard of commercial pullulan (Sigma-Aldrich, St. Louis, Missouri, United States) was treated as the same procedure.

### Purification and diluted-acid hydrolysis of Ca-PMA

The culture after 7 days obtained from the PMA production medium (50 ml) was centrifuged at 10,000 × g and 4°C for 5 min to remove cells. Firstly, 5 ml of pure methanol was added to 10 ml of supernatant to selectively remove exopolysaccharide (EPS) as a precipitate. After the removal of EPS, pure methanol was again added to the supernatant and then incubated at 4°C for 12 h. The Ca-PMA precipitate was obtained by centrifuging at 10,000 × g and 4°C for 5 min and was thoroughly dissolved in 5 ml deionized water. Again 15 ml pure methanol was added, followed by incubation at 4°C for 12 h. The above procedures were repeated three times. The final precipitated Ca-PMA was desiccated using a vacuum pump. The chemical hydrolysis of the purified Ca-PMA was carried out with 0.5 M H_2_SO_4_ in a sealed glass tube at 90°C [93].

#### Analysis of hydrolysis products of pullulan and Ca-PMA

The hydrolysates of pullulan and Ca-PMA were analyzed via a DIONEX UltiMate 3000 HPLC System (Thermo Scientific, Waltham, MA, USA). The products after hydrolysis were separated on a Metab-AAC column (300 × 7.8 mm column, ISERA, Düren, Germany). The HPLC running conditions were as follows: the elution was performed with 5 mM H_2_SO_4_ at a flow rate of 0.6 ml/min with a temperature of 40°C. For detection, a SHODEX RI-101 detector (Showa Denko Europe GmbH, München, Germany) and a DIONEX UltiMate 3000 Variable Wavelength Detector set to 210 nm were used. The standard chemicals of maltotriose, glucose, L-malic acid, and the racemic mixture of D- and L-malate were bought from Sigma-Aldrich (St. Louis, Missouri, United States) and used.

#### Analysis of fungal ploidy using flow cytometry

The ploidy type was determined using flow cytometry according to a published method by Todd et al. [94]. In previous publications, the strain *A. melanogenum* EXF-7946 was shown to have a haploid genome, while *A. melanogenum* EXF-8492 showed to be diploid [95]. These strains were used as haploid and diploid references during the analyses.

## Results and discussion

### Whole-genome sequencing metrics and analysis of repetitive sequences

The genomic DNA of the yeast-like fungus *A. pullulans* NRRL 62031 was previously sequenced using the Illumina HiSeq platform with an average depth of 245-fold [66]. As displayed in Table 1, the 25.05-Mb draft genome sequence was assembled into 209 scaffolds ranging from 1,004 bp to 1,881,877 bp. The GC content is 50%. The average length of scaffolds was 120,997 bp with an N50 of 1,223,087 bp and N75 of 851,637 bp. This N50 value is significantly higher than the average N50 value (74,165) of 50 *A. pullulans* strains [63], suggesting that the genome assembly is of high quality and suitable for subsequent analysis. A total of 9,241 protein-coding genes were predicted, with an average length of 1,542 bp, making up 56% of the whole genome. The genome carries 55 rRNA, 235 tRNA, and 47 other non-coding RNA genes. These findings are consistent with previously published annotations for *A. pullulans* strains, which reported around 250–300 predicted tRNA genes and around 20–60 rRNA genes [62, 96]. In 2019, fifty genomes of *A. pullulans* isolated from different habitats were assembled at contig levels via the Illumina NextSeq platform [63]. The genome size and GC content of *A. pullulans* NRRL 62031 were slightly smaller than the average assembly size and GC content of all fifty *A. pullulans* strains (28 ± 13 Mbp, 51%), while the number of predicted genes (9,241) of *A. pullulans* NRRL 62031 was lower than the mean value (10,646) of all fifty *A. pullulans* strains [63]. In 2021, the average genome size and gene number of forty-nine *A. melanogenum* strains were 41 ± 10 Mbp and 18,745, respectively, which is much higher than that of the sequenced *A. pullulans* NRRL 62031. The large average genome size and the high deviation from the mean size of *A. melanogenum* strains are due to the fact that more than half of them are diploid strains, thus having greatly differing genome sizes [95]. Their mean GC value (50%) is close to that of *A. pullulans* NRRL 62031 [95]. More recently, a total of eight *A. subglaciale* strains were sequenced at the whole genome level [97, 98]. It was presented that the average size, GC content, and predicted number of genes of the sequenced *A. subglaciale* strains is 26 ± 0.5 Mbp, 51%, and 9,457, respectively, which is similar to those of *A. pullulans* NRRL 62031 [97]. Table 2 shows the mean value of genome assembly metrics for the sample sets of *A. pullulans*, *A. melanogenum*, and *A. subglaciale*, respectively.

**Table 1:** General genome statistics of *A. pullulans* NRRL 62031

Characteristics	Value
Length (bp)	25,051,135
Scaffolds	209
GC content (%)	50.07
N50 length (bp)	1,223,087
N75 length (bp)	851,637
Maximum length (bp)	1,881,877
Minimal length (bp)	1,004
Average length (bp)	120,997.04
The average depth	244.86 ×
Protein-coding gene number	9,241
Average length of predicted protein-coding genes (bp)	1541.85
Coding regions (bp)	14,248,224
rRNA	55
tRNA	235
Other ncRNA	47
KEGG assignment	5,757
GO assignment	5,238
KOG assignment	5,318
NCBI-NR assignment	9,153
Swiss-Prot assignment	6,595
CAZymes assignment	946
Pfam assignment	6,995
TCDB assignment	308

**Table 2:** The average genome assembly metrics from sequencing projects including the sequences of 50 *A. pullulans* strains [63], 49 *A. melanogenum* strains [95], and 8 *A. subglaciale* strains [97]

	*A. pullulans*	*A. melanogenum*	*A. subglaciale*
Genome assembly size (Mb)	28.04 (± 1.03)	41.43 (± 10.25)	25.97 (± 0.54)
Number of contigs (n)	1,629 (± 1,523)	N/A	N/A
N50 length (bp)	74,165 (± 88,815)	N/A	N/A
GC content (%)	50.65 (± 0.14)	50.04 (± 0.46)	50.66 (± 0.08)
Gene number (n)	10,646 (± 238)	18,745 (± 6,405)	9,457 (± 343)
Gene average length (bp)	1,564 (± 50)	1,378 (± 232)	1,607 (± 16)
CDS total length (% of genome)	54.91 (± 1.28)	53.18 (± 2.47)	54.20 (± 1.18)
CDS total length (Mbp)	15.40 (± 0.66)	22.01 (± 5.52)	14.08 (± 0.38)

*CDS* coding sequence, *N/A* not available, *N50* the sequence length of the shortest contig at 50% of the total assembly length

The genome size of 25.05 Mb suggests that the genome is haploid. In a study sequencing 49 *A. melanogenum* strains, 30 strains were identified to be diploid with a genome size ranging around 50 Mb, while the clearly haploid strains had genome sizes below 30 Mb [95]. Apart from the genome size, flow cytometry was used to determine the ploidy. The ploidy of *A. pullulans* NRRL 62031 was determined by comparing the fluorescent intensity of the stained DNA to the intensity of the references. *A. pullulans* NRRL 62031 showed a fluorescence pattern similar to the haploid reference, strongly indicating that the strain carries a haploid genome (Fig. S1).

The repetitive DNA sequences in the genome are classified into two categories according to their complexity and multitude: interspersed repeats (IRs), scattered throughout the genome, and tandem repeats (TRs), repeated in an organized pattern in one localized region [99, 100]. The tandem repeats mainly include microsatellites, simple sequence repeats (2–6 bases as a repeating unit), minisatellites (10–100 bases of long sequences as a repeating unit), and others [101]. As shown in Table S2, the genome of *A. pullulans* NRRL 62031 contained 54 small RNAs, 2,615 simple repeats, and 387 sequences with low complexity. However, no satellite RNA sequences were found. Most of the interspersed repeats are transposable elements (TEs) [102]. The genome of *A. pullulans* NRRL 62031 did not contain short interspersed nuclear elements, long interspersed nuclear elements, and long terminal repeats. However, 271 interspersed repeats were not classified. It is worth noting that no DNA transposons were identified in the genome of *A. pullulans* NRRL 62031. All these results (small size, low amount of repetitive sequences, no transposons) suggest that the genome of *A. pullulans* NRRL 62031 is likely stable. In contrast, the chromosomes of *A. melanogenum* TN3-1 isolated from honey harbor a high level of transposable elements [103].

### Comparative genome analyses suggest a reclassification of *A. pullulans* NRRL 62031

**Fig. 1:**
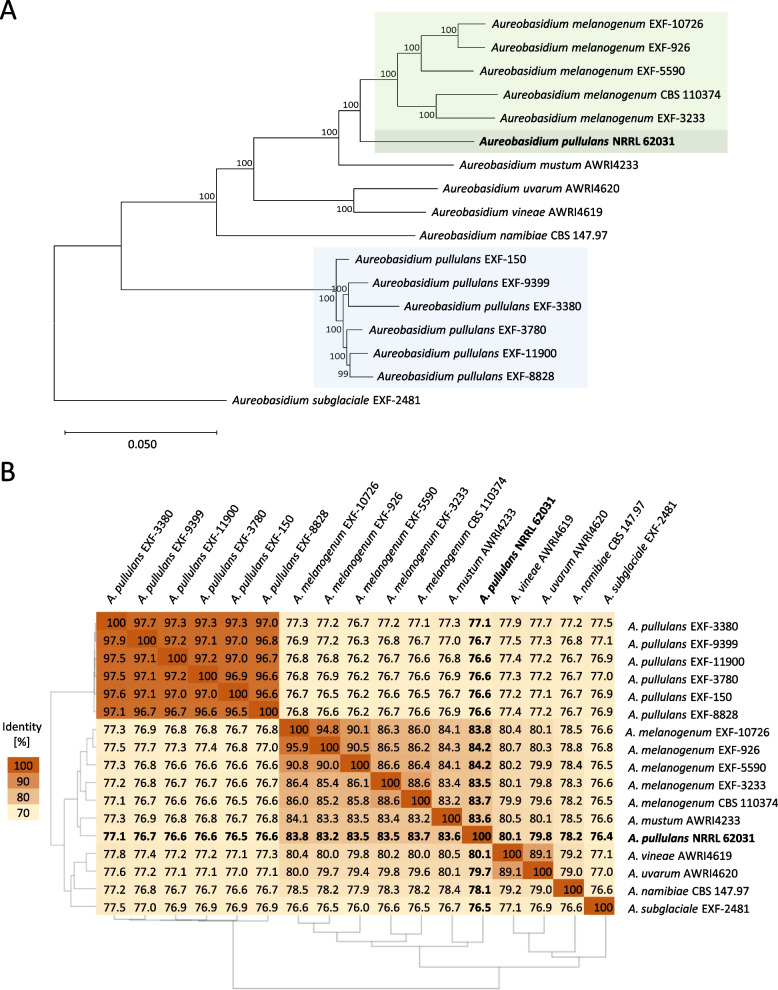
Phylogenetic tree and Pairwise Average Nucleotide Identity (ANI) analysis of selected *Aureobasidium* strains. The comparative genome analysis of *A. pullulans* NRRL 62031 and 16 publicly available genomes of members of the genus *Aureobasidium* was performed using the comparative genomics tool EDGAR 3.2. **A** The phylogenetic tree construction was based on the 5,415 core genes. Within the EDGAR tool, the Shimodaira-Hasegawa (SH)-like local support values were computed using FASTtree. **B** The ANI was calculated as the mean identity of all BLASTN matches. The darker the shade of orange, the higher the similarity between two strains

To deduce the phylogeny of *A. pullulans* NRRL 62031, a phylogenetic tree was computed with 16 publicly available genome sequences of *Aureobasidium* strains from various species and habitats (Fig. 1A). Detailed information on these 16 strains is displayed in Table S1. The strains were selected to cover different *Aureobasidium* species fully sequenced so far. Specifically, six *A. pullulans* and five *A. melanogenum* strains were included, originating from different isolation sites to represent a high habitat diversity. Using this data set made it possible to account for strain variability. A deliberate decision was made not to select a larger number of strains for this study for reasons of simplicity. The phylogenetic tree was constructed with the comparative genomics platform EDGAR 3.2 and is based on 5,415 core genes shared by 17 *Aureobasidium* strains [86, 104]. The phylogenetic tree showed that *A. pullulans* NRRL 62031 clustered with *A. melanogenum* strains, separate from the clade classified as *A. pullulans*. This indicates that the strain might belong to the species *A. melanogenum*. However, the strain did not directly group with one of the *A. melanogenum* strains.

For additional information regarding the phylogenetic classification of the sequenced strain, an average nucleotide identity analysis (ANI, Fig. 1B) was performed to determine the nucleotide-level genomic similarity between the genomes. ANI analysis is commonly used to define species in prokaryotes, with a 95% cut-off proposed for species boundaries [105]. However, this doesn't directly apply to fungi due to limited ANI data for fungi. The ANI values between the *Aureobasidium* species varied from 77 to 98%. It is striking that the strains of the species *A. pullulans* have a high similarity (> 97%), whereas the strains of the species *A. melanogenum* differ more strongly, with a similarity between 86 and 96%. A study by Onetto et al. [19] also showed high similarity among *A. pullulans* strains, with ANI values ranging from 96.5% to 99.9%. The strain NRRL 62031 showed a similarity of approximately 77% to the considered *A. pullulans* species included in this analysis, reinforcing the conclusions drawn from the phylogenetic tree analysis and suggesting that its classification as *A. pullulans* is incorrect. A higher similarity could be observed between NRRL 62031 and the *A. melanogenum* strains included in the present study with ANI values between 83 and 84%.

Overall, the reclassification of *A. pullulans* NRRL 62031 to *A. melanogenum* seems reasonable. In fact, the phylogeny of *A. pullulans* NRRL 62031 was originally confirmed through DNA sequence analyses of the internal transcribed spacer region (ITS) and β-tubulin (BT2) in 2012 [40]. The ITS region is the official DNA barcoding marker for species-level identification of fungi, while the BT2 region is less conserved compared to the ITS and thus provides information on the subspecies level [106, 107].

The redefinition of *A. pullulans* and *A. melanogenum* as separate species was only done in 2014 [108]. Although, a classification into the varieties *A. pullulans* var. *pullulans* and *A. pullulans* var. *melanogenum* existed before [11]. However, the availability of genome sequence data from *Aureobasidium* strains was low at the time and has increased rapidly in recent years. A current BLASTN search of the ITS and BT2 sequences from *A. pullulans* NRRL 62031, utilizing a higher number of whole genome sequences from *A. pullulans* and *A. melanogenum* strains also demonstrates that *A. pullulans* NRRL 62031 is more likely classified within the species *A. melanogenum*. Specifically, when using the ITS as the query, the first 50 hits sorted by max score contain 49 *A. melanogenum* strains and one not classified *Aureobasidium* strain. For the BT2 sequence as query, the first 50 hits also contain 49 *A. melanogenum* strains and one *A. pullulans* strain.

Based on the data presented, a reclassification of *A. pullulans* NRRL 62031 should be considered. However, this would require a more comprehensive analysis.

### Genomic functional annotation reveals high metabolic and catalytic potential

The gene annotation of *A. pullulans* NRRL 62031 was carried out by comparing the 9,241 predicted protein sequences with the protein sequences from multiple public databases: Kyoto encyclopedia of genes and genomes (KEGG) (5,757 matching genes), gene ontology (GO) (5,238 matching genes), eukaryotic orthologous groups (KOG) (5,318 matching genes), non-redundant (NCBI-NR) (9,153 matching genes), Swiss-Prot (6,596 matching genes), carbohydrate-active enzymes database (CAZymes) (946 matching genes), the protein families database (Pfam) (6,995 matching genes), and transporter classification database (TCDB) (308 matching genes) as shown in Table 1.

**Fig. 2:**
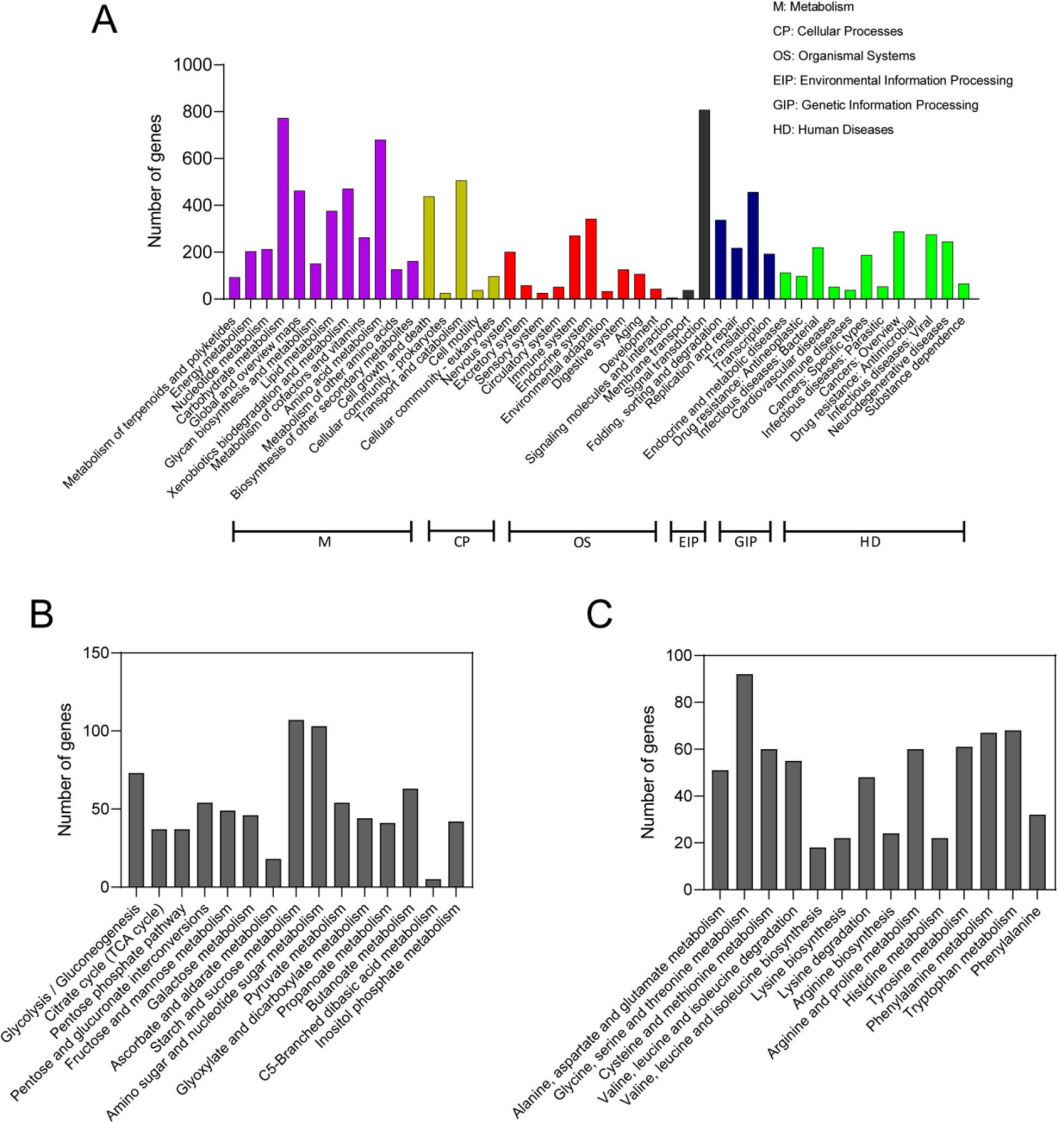
The function annotation of the predicted protein-coding genes in *A. pullulans* NRRL 62031 based on the Kyoto encyclopedia of genes and genomes (KEGG) database. **A** The overview of KEGG classification of putative protein-coding genes. It can be divided into the categories of metabolism, cellular processes, organismal systems, environmental information processing, genetic information processing, and human diseases. **B** KEGG classification of carbohydrate metabolism. **C** KEGG classification of amino acid metabolism

The KEGG pathway classification of the predicted genes in *A. pullulans* NRRL 62031 was displayed in Fig. 2A. Specifically, the predicted protein-encoding genes were categorized into six main classes: metabolism (3,976 genes), cellular processes (1,104 genes), organismal systems (1,263 genes), environmental information processing (852 genes), genetic information processing (1,206 genes), and human diseases (1,639 genes) [109]. Some genes may encode proteins with similar functions to human disease-related proteins. These similarities are based on proteins’ sequence, structure, and domain. These genes may not be directly involved in the occurrence of human diseases [71, 110, 111]. The alignment against the KEGG database suggested that protein-encoding genes mainly belonged to the metabolism. Moreover, the categories carbohydrate metabolism (773 genes) and amino acid metabolism (680 genes) comprised more genes than other categories in the metabolism class, indicating that *A. pullulans* NRRL 62031 has pronounced catabolic and anabolic activities. Specifically, in *A. pullulans* NRRL 62031, most of the genes were represented in starch and sucrose metabolism, followed by amino sugar and nucleotide sugar metabolisms, glycolysis/gluconeogenesis, butanoate metabolism, and other sub-terms involved in the primary intracellular metabolism such as TCA cycle, glyoxylate shunt, and pentose phosphate pathway (Fig. 2B). Concerning the term amino acid metabolism, most genes were concentrated in glycine, serine, and threonine metabolisms, followed by tryptophan, phenylalanine metabolisms, and other amino acid-related sub-terms (Fig. 2C). Moreover, it was highly intriguing that a substantial number of genes were found in the signal transduction category (Fig. 2A). It can be speculated that *Aureobasidium* spp. have evolved to feature various signaling systems for the utilization of many substrates and the adaptation to high-stress environments [112].

The GO database is a community-based bioinformatics resource that classifies gene product functions by the use of structured and controlled vocabularies [113]. The gene products aligned to the GO database are divided into three major classes: molecular function, biological process, and cellular component [114]. A majority of genes (2,577) were assigned to catalytic activity (GO:0003824), a subclass of molecular function, primarily including hydrolases, lyases, oxidoreductases, transferases, and cyclases in *A. pullulans* NRRL 62031 (Fig. S2). The second largest share of genes (2,540) was represented in binding (GO:0005488), also a subclass of molecular function, in *A. pullulans* NRRL 62031 (Fig. S1). Some enzymes that take part in the hydrolysis of plant polysaccharides were annotated, such as cellulose-binding (GO:0030248) enzymes, starch-binding (GO:2001070) enzymes, and others. In addition, 301 representatives of the zinc ion binding category (GO:0008270) were assigned to the GO database (data not shown). It is reported that zinc-binding proteins are associated with transcriptional regulation and zinc chelation in fungal cells [115]. The protein-encoding genes of the biological process class were predominantly allocated to metabolic process (GO:0008152, GO:0044236, GO:0044710) and cellular process (GO:0009987, GO:0008151, GO:0044763, GO:0050875), which included 2,067 and 1,127 genes, respectively. They were mainly composed of carbohydrate metabolic process (210 genes), nitrogen compound metabolic process (18 genes), lipid metabolic process (47 genes), and cellular metabolic process (12 genes) (data not shown).

The KOG database is a repository of eukaryotic proteins. It can be used to identify orthologous genes from the COG (clusters of orthologous groups) database [73, 116]. 5,318 genes were aligned to the KOG database, comprising 58% of the total protein-coding genes. For the metabolism class, the protein-encoding genes aligned to the terms carbohydrate transport and metabolism (342 genes), amino acid transport and metabolism (325 genes), energy production and conversion (324 genes), and lipid transport and metabolism (318 genes). Additionally, 258 genes were assigned to secondary metabolite biosynthesis, transport, and catabolism (Fig. S3). This indicates that *A. pullulans* NRRL 62031 seems to have considerable potential to biosynthesize a large number of unknown value-added compounds. It was of interest to note that 398 genes were predicted to be relevant to signal transduction mechanisms, the second largest group next to posttranslational modification, protein turnover, and chaperones in the class of intracellular processes, which implied that this strain possesses versatile signaling transduction pathways (Fig. S3). It was in line with the previous KEGG annotation that a large number of genes were assigned to the signal transduction term.

### Carbon substrate dependent growth kinetics

Microorganisms that have a broad growth substrate range are attractive for biorefineries. The growth rate of *A. pullulans* NRRL 62031 on minimal medium supplemented with 2% (w/v) of diverse carbon sources was measured. The carbon sources were selected to represent the diverse substrates in biorefineries, including saccharides (glucose, xylose, fructose, galactose, sucrose, lactose, maltose, cellobiose, and starch), polyols/alcohols (xylitol, mannitol, sorbitol, glycerol, ethanol, methanol, and butanol), organic acids (citric acid, fumaric acid, succinic acid, formic acid, and acetic acid), and plastic monomers (1,4-butanediol, terephthalic acid, adipic acid, and ethylene glycol).

**Fig. 3:**
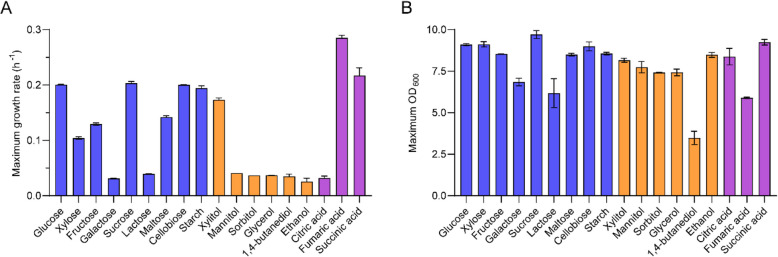
The growth properties of *A. pullulans* NRRL 62031 on saccharides (blue bars), polyols (orange bars), and organic acids (purple bars). **A** The maximum growth rate (h^−1^) of *A. pullulans* NRRL 62031 grown on different carbon sources. **B** The maximal OD_600_ value of *A. pullulans* NRRL 62031 grown on different carbon sources. The error bars show deviation from the mean values based on two biological replicates

The highest growth rate of *A. pullulans* NRRL 62031 was 0.29 h^−1^ measured on fumaric acid. Growth on sucrose, cellobiose, succinic acid, and starch was around 0.20 h^−1^, which was highly similar to growth on glucose, followed by growth on xylitol, maltose, fructose, and xylose. However, the cell growth of *A. pullulans* NRRL 62031 on galactose, lactose, mannitol, sorbitol, glycerol, citric acid, 1,4-butanediol, and ethanol showed similar growth rates at the lowest level among all tested carbon sources. Interestingly, fumaric acid, succinic acid, and citric acid as important metabolic intermediates in the TCA (tricarboxylic acid) cycle had different influences on the maximum growth rate of *A. pullulans* NRRL 62031 (Fig. 3A). It was reported that the maximal growth rate of *A. pullulans* NRRL 62042 is 0.18 h^−1^ in an optimized minimal polyol lipid production medium using sucrose as the sole carbon source [36]. This rate is slightly lower than the maximal growth rate (0.20 h^−1^) on sucrose in this study. It might be because more carbon flux flows to the synthesis of polyol lipids in the reported production medium.

The final cell densities of *A. pullulans* NRRL 62031 grown on the respective carbon sources did not show striking differences from those grown on glucose except for galactose, lactose, fumaric acid, and 1,4-butanediol (Fig. 3B). The growth assay results revealed that *A. pullulans* NRRL 62031 could grow on starch as the sole carbon source, and its maximum growth rate and maximum OD_600_ value were very close to that of glucose (Fig. 3). It demonstrated that *A. pullulans* NRRL 62031 could grow very well on starch due to the high secretion of starch-degrading enzymes and hence the depolymerization was not the rate-limiting step. However, no growth of *A. pullulans* NRRL 62031 was detected on methanol, butanol, formic acid, acetic acid, ethylene glycol, terephthalic acid, and adipic acid (data not shown), which could be generally explained by two reasons: One reason is that some substrates are toxic to the microbes even though some indigenous enzymes can degrade the chemicals. For example, formic acid negatively affects the cell growth of *A. pullulans* NRRL 62031 even though one gene copy (g3023) coding for a formate dehydrogenase (EC 1.2.1.2) was present. Another reason is that no gene products were found to be involved in the degradation pathways of the respective substrates. For example, no methanol oxidase (EC 1.1.3.31) was found in *A. pullulans* NRRL 62031 for methanol degradation.

The genomic features in *A. pullulans* NRRL 62031 were analyzed to identify genes relevant to the established growth capacities. One gene (g6591) encoding xylose reductase (EC 1.1.1.307), four genes (g801, g3766, g6680, and g6851) encoding NAD^+^-dependent xylitol dehydrogenases (EC 1.1.1.9), three genes (g5975, g6046, and g8503) encoding xylulokinases (EC 2.7.1.17) were found in *A. pullulans* NRRL 62031. It suggests that this strain could utilize xylose through the reductase/xylitol dehydrogenase (XR/XDH) pathway where xylose reductase reduces xylose to xylitol, and then xylitol is oxidized to xylulose by NAD^+^-dependent xylitol dehydrogenase. Xylulose is further phosphorylated to xylulose-5-phosphate and subsequently enters the pentose phosphate pathway [117–119]. *A. pullulans* NRRL 62031 possessed four genes (g1443, g3017, g7938, and g7939) coding for β-fructofuranosidases (invertases) (EC 3.2.1.26), which are the key enzymes for sucrose utilization [120]. β-Glucosidase cleaves β−1,4 bonds linking two glucose or glucose-substituted molecules like in cellobiose [121]. β-Glucosidase is a mostly extracellular hydrolase, which has the potential to be used in various biotechnological applications such as biofuel production, flavor enhancement, and oligosaccharides synthesis [122]. A total of 17 genes (g588, g1124, g1920, g3053, g3818, g4004, g4018, g4311, g5474, g5886, g6391, g6683, g7245, g7479, g7664, g8489, and g8770) encoding β-glucosidases (EC 3.2.1.21) were present in *A. pullulans* NRRL 62031.

Starch can be hydrolyzed into glucose by α-amylase and glucoamylase [123]. In *A. pullulans* NRRL 62031, five genes (g1316, g3298, g3502, g6635, and g8572) encoded α-amylases (EC 3.2.1.1) and eleven genes (g1122, g1315, g3274, g4333, g4383, g4908, g5111, g5826, g6265, g8897, and g8936) coded for glucoamylases (EC 3.2.1.3). Hult and Gatenbeck proposed that mannitol 2-dehydrogenase (Mdh) is a key mannitol degradation enzyme [124]. Only one gene (g7319) was predicted to be an NAD^+^-dependent mannitol 2-dehydrogenase (EC 1.1.1.67), but NADP^+^-dependent mannitol 2-dehydrogenase (EC 1.1.1.138) was absent in *A. pullulans* NRRL 62031. In eukaryotic cells, it was proposed that an NAD^+^-dependent sorbitol dehydrogenase (EC 1.1.99.21) oxidizes D-sorbitol to D-fructose, which is then phosphorylated to D-fructose-6-phosphate [125]. However, no putative NAD^+^-dependent sorbitol dehydrogenase-coding genes were found in *A. pullulans* NRRL 62031. It is likely that other promiscuous dehydrogenases, with a wide spectrum of substrates were responsible for the sorbitol dehydrogenation. It was proposed that glycerol in fungal cells is first phosphorylated by glycerol kinase (EC 2.7.1.30) to glycerol-3-phosphate catalyzed, which is then catalyzed to dihydroxyacetone phosphate mediated by mitochondrial glycerol-3-phosphate dehydrogenase (EC 1.1.5.3) [126, 127]. It was found that three genes (g1846, g3798, and g9231) coded for glycerol kinases and five genes (g170, g1552, g5709, g7927, and g8363) coded for mitochondrial glycerol-3-phosphate dehydrogenases in *A. pullulans* NRRL 62031. It was reported that two different species of *Aureobasidium* spp. contain one mitochondrial glycerol-3-phosphate dehydrogenase and two copies of glycerol kinases [128]. In *S. cerevisiae*, the alcohol dehydrogenase 2 (Adh2) catalyzes the conversion of ethanol to acetaldehyde, which is then converted to acetate catalyzed by acetaldehyde dehydrogenases (Ald1-Ald7) [129]. A total of 13 genes (g302, g442, g1170, g2835, g3554, g4445, g5777, g6376, g6503, g6929, g7193, g7993, and g8448) encoded alcohol dehydrogenase (EC 1.1.1.1). However, no gene products were found to be NAD(H)^+^-dependent acetaldehyde dehydrogenase (EC 1.2.1.3/EC 1.2.1.4) in *A. pullulans* NRRL 62031. It can be explained by the existence of aldehyde dehydrogenases (EC 1.2.1.5), which play a role in the conversion from acetaldehyde to acetate. Indeed, two genes (g5063 and g6568) coded for aldehyde dehydrogenases in *A. pullulans* NRRL 62031. Aldehyde dehydrogenases belong to a superfamily of enzymes that oxidize a wide spectrum of endogenous and exogenous aldehydes to their corresponding carboxylic acids [130, 131]. 1,4-butanediol, a typical plastic monomer, is released during the depolymerization of polyurethanes. Two genes (g7513 and g7678) encoding (R,R)-butanediol dehydrogenases, could be attributed to 1,4-butanediol metabolism in *A. pullulans* NRRL 62031. It was found that the alcohol dehydrogenases PedE and PP_2049 in *Pseudomonas putida* KT2440 are essential for growth on 1,4-butanediol as the sole carbon source [132]. However, no protein homologs of PedE and PP_2049 were identified in *A. pullulans* NRRL 62031. Three types of organic acids (citric acid, fumaric acid, and succinic acid) tested are intermediates of the TCA cycle. Indeed, two citrate synthase-encoding genes (g169 and g5952) (EC 2.3.3.1), one aconitate hydratase-encoding gene (g2365) (EC 4.2.1.3), two isocitrate dehydrogenase-encoding genes (g2930 and g4813) (EC 1.1.1.41), one ketoglutarate dehydrogenase-encoding gene (g3556) (EC 1.2.4.2), three succinate-CoA synthetase-encoding genes (g1802, g3979, and g5526) (EC 6.2.1.4), five succinate dehydrogenase-encoding genes (g1547, g4148, g7599, g8140, and g9066) (EC 1.3.5.1), two fumarate hydratase-encoding genes (g6753 and g7477) (EC 4.2.1.2), and two malate dehydrogenase-encoding genes (g4110 and g5765) (EC 1.1.1.37) were present in *A. pullulans* NRRL 62031.

All predicted protein sequences against the TCDB (transporter classification database) showed that a total of 308 genes in *A. pullulans* NRRL 62031 might code for membrane transport proteins. In the present study, a total of 25 genes coded for permeases, allowing the diffusion of some molecules such as amino acids, purine, quinate, nitrate, sulfate, and so on. The specific transporters responsible for simple sugars tested including glucose, fructose, xylose, and galactose may be attributed to five monosaccharide transporters encoding genes (g6, g9102, g2967, g3956, and g4860), two glucose/xylose: H^+^ symporter encoding genes (g715 and g9052), one high-affinity glucose transporter encoding gene (g6826). In addition, one gene (g1016) coded for a glycerol uptake facilitator might be responsible for the assimiliation of glycerol in *A. pullulans* NRRL 62031. The ATP-binding cassette (ABC) and major facilitator superfamily (MFS) transporters in fungi are associated with the defense against natural toxic compounds [133, 134]. Four genes (g1265, g6158, g1527, and g3642) coded for ABC transporters, while only one gene (g2926) coded for a MFS transporter. Aquaglyceroporin, as a subgroup of aquaporin, can facilitate specific passive transport of uncharged small solutes such as water, glycerol, and other polyols [135]. Three aquaporins encoding genes (g5088, g5147, and g6125) were found in *A. pullulans* NRRL 62031. It could be speculated that xylitol, mannitol, sorbitol, glycerol, and 1,4-butanediol may be transported across the cytoplasmic membrane by aquaporins.

In summary, these results suggest that the black yeast *A. pullulans* NRRL 62031 can utilize a variety of carbon sources, including saccharides, polyols, organic acids, and 1,4-butanediol. Key enzymes and transporters responsible for their respective utilization were identified in the genome of *A. pullulans* NRRL 62031.

### Hydrolysis potential regarding plant polysaccharides

**Fig. 4:**
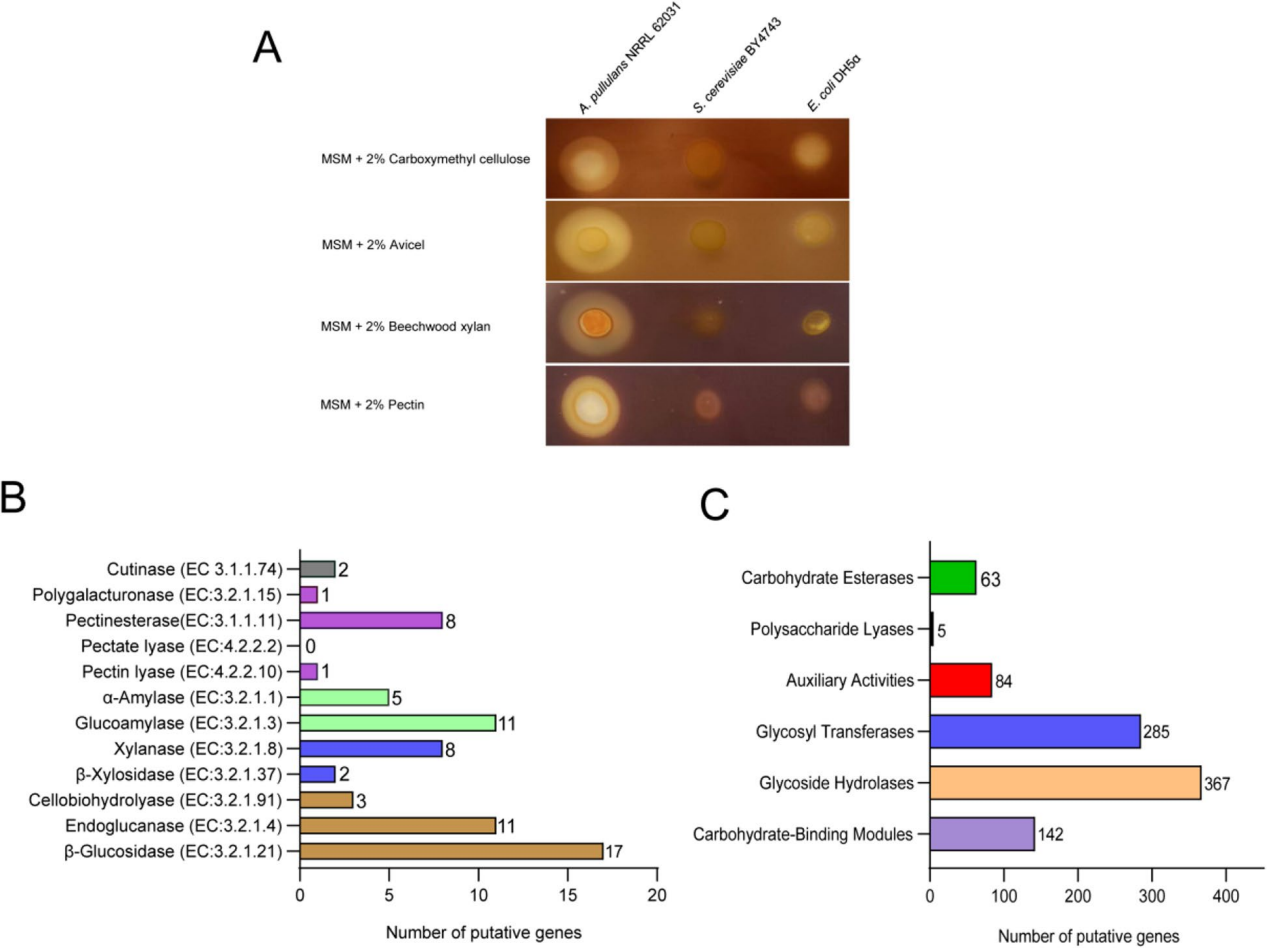
Physiological and genetic evidence about biopolymer hydrolysis abilities of *A. pullulans* NRRL 62031. **A** Results of the halo visualization assay on minimal agar plates with 2% (w/v) carboxymethyl cellulose, Avicel, beechwood xylan, and pectin. **B** KEGG term classification of possible hydrolytic enzymes regarding plant biomass depolymerization for which the respective genes were found in the genome of *A. pullulans* NRRL 62031. **C** CAZy term classification of putative hydrolytic enzymes related to the decomposition of plant biomass in *A. pullulans* NRRL 62031

Using cheap substrates, such as agricultural side or waste streams, for biotechnological production is highly relevant for reducing biomanufacturing costs and increasing sustainability. The capability for broad-spectrum saccharification of plant biomass was indicated by various genes encoding putative hydrolytic enzymes based on the KEGG annotations. As displayed in Fig. 4B, 17 β-glucosidases (EC 3.2.1.21), eleven endoglucanases (EC 3.2.1.4), and three cellobiohydrolases (EC 3.2.1.91) predicted in *A. pullulans* NRRL 62031 might contribute to cellulose utilization. Eight genes encoding xylanases (EC 3.2.1.8) and two genes encoding β-xylosidases (EC 3.2.1.37) were related to xylan degradation. The starch hydrolytic enzymes included five α-amylases (EC 3.2.1.1) and eleven glucoamylases (EC 3.2.1.3). One gene coding for a polygalacturonase (EC 3.2.1.15), eight genes coding for pectinesterases (EC 3.1.1.11), and one gene coding for a pectin lyase (EC 4.2.2.10) were implicated with the hydrolysis of pectin. Two genes coding for cutinases (EC 3.1.1.74) were likely responsible for the decomposition of cutin. The CAZy database [136] (http://www.cazy.org/) displays the families of structurally related catalytic and carbohydrate-binding modules of enzymes that build, modify, and break down glycosidic bonds for a significant number of biological functions. Additionally, CAZy is a knowledge-based resource dedicated to coupling the sequence information, the specificity, and the 3D structural features of CAZymes [136, 137]. With its annotation information, the proteins were categorized into glycoside hydrolases (GHs), glycosyltransferases (GTs), polysaccharide lyases (PLs), carbohydrate esterases (CEs), carbohydrate-binding modules (CBMs), and auxiliary activities (AAs) [138, 139]. A total of 946 genes in *A. pullulans* NRRL 62031 were predicted to be involved in the hydrolysis and modification of oligo- and polysaccharides, making up about 10% of all protein-encoding genes (9,241). This feature is consistent with the fact that *A. pullulans* NRRL 62031 was collected from lignocellulosic material (a leaf) in Thailand [40].

The class of glycoside hydrolases (GHs) constitutes the highest proportion (39%), followed by glycosyltransferases (GTs) (30%), carbohydrate-binding modules (CBMs) (15%), auxiliary activities (AAs) (8.9%), carbohydrate esterases (CEs) (6.7%), and polysaccharide lyases (PLs) (0.5%) (Fig. 4C). It was reported that the number of GH family enzymes in *A. pullulans* isolated from grape juice occupied the highest among the CAZy families [19]. GHs enzymatically cleave the glycosidic bonds between two or more carbohydrates or between a carbohydrate and a non-carbohydrate moiety [136]. Other strains of *A. pullulans* also have a large number of GHs. For instance, the enzyme-encoding genes in *A. pullulans* AWRI4231 and *A. pullulans* EXF-150 corresponding to the GH class constitute 68% and 56% of their respective secreted carbohydrate-active enzymes, respectively [4, 19]. The GT class makes up the second largest proportion (30%) among the six classes of CAZymes. The class of GTs is involved in the biosynthesis of natural glycans, transferring a sugar moiety from the donor to acceptor substrates such as oligosaccharides, polysaccharides, lipids, and proteins [140]. CBMs have been found in many polysaccharide-degrading enzymes, including cellulases and xylanases [141]. A total of 142 enzyme-encoding genes in *A. pullulans* NRRL 62031 were assigned to the families of CBMs.

PLs mainly degrade glycosaminoglycans and pectin [142]. However, only five genes were identified as encoding PL enzymes. This number is similar in other *A. pullulans* strains. For example, the isolates *A. pullulans* AWRI4231 and *A. pullulans* EXF-150 had ten and eleven PL enzymes, respectively [4, 19]. The family of CE5 is responsible for cleaving ester bonds in cutin to release cutin monomers [142]. A total of eight enzyme-encoding genes belong to the CE5 family (data not shown). The class of AA enzymes assists other CAZymes in degrading a complex substrate. A total of 84 genes fell into the AA class in the CAZy database (Fig. 4C).

Furthermore, the hydrolysis potential of *A. pullulans* NRRL 62031 on cellulose, beechwood xylan, and pectin was tested through the halo visualization assay [90, 143]. As displayed in Fig. 4A, the formed discernible halos around a single colony were observed on agar plates containing 2% (w/v) cellulose, beechwood xylan, or pectin, suggesting that *A. pullulans* NRRL 62031 can extracellularly secrete cellulolytic, xylanolytic, and pectinolytic enzymes to depolymerize the respective polymers. In contrast, no clear zones were observed for *S. cerevisiae* BY4743 and *E. coli* DH5α, serving as negative controls.

In this study, 10 genes (g1377, g1382, g3283, g4053, g4068, g4594, g5079, g7760, g7766, and g8628) encoding phenol 2-monooxygenases and one gene (g7759) encoding catechol dioxygenase were present in *A. pullulans* NRRL 62031. It has been reported that these two enzymes are involved in ring hydroxylation of adjacent carbon atoms and ring cleavage of the formed catecholic intermediates [144, 145]. Therefore, this strain might be able to biodegrade aromatic pollutants such as phenol, biphenol, benzoate, catechol, and phenylpropanoid. Moreover, *A. pullulans* NRRL 62031 contained genes encoding different types of lipases such as phospholipases, triglyceride lipases, triacylglycerol lipases, esterases, and lysophospholipases (data not shown). Lipase activities have been linked to the significant reduction of hydrocarbons in contaminated soil areas [146]. It was shown that *A. pullulans* NRRL 62031 has a detectable lipase activity at 0.15 U/ml [147]. Laccases are relevant for lignin degradation and bioremediation of dyes and toxic materials [58, 148]. However, only one gene (g4822) encoding a laccase was present in *A. pullulans* NRRL 62031.

All these findings suggest that *A. pullulans* NRRL 62031 features hydrolytic activities on agricultural biomass such as cellulose, xylan, pectin, and starch. In addition, it might be helpful to decompose cutin, aromatic compounds, waste oils, lignin, and other environmental contaminants. To fully exploit this potential, further research is needed to determine the specific conditions required for the production of these enzymes in *A. pullulans* NRRL 62031.

### Mining the genome for biosynthetic genes

In fungi, secondary metabolic pathways are often encoded by biosynthetic gene clusters (BGCs). A BGC is composed of two or more physically consecutive genes that participate in an anabolic pathway for producing a specific metabolite [149, 150].

The antiSMASH (antibiotics & secondary metabolite analysis shell) fungal pipeline version 7.1.0 was applied to identify putative BGCs in *A. pullulans* NRRL 62031, resulting in 23 BGCs. Specifically, they contained four T1PKSs (type I polyketide synthases), one T3PKS (type III polyketide synthase), five NRPSs- (non-ribosomal peptide synthases) like, five terpene, one betalactone, one NAPAA (non-alpha poly-amino acid), two fungal-RiPP- (fungal unspecified ribosomally synthesized and post-translationally modified peptide product) like, one T1PKS/NRPS-like, one NRPS/betalactone, one NRP-metallophore/NRPS, one T1PKS/NRPS-like/indole BGCs. A total of three T1PKS BGCs were aligned to the recorded BGCs of yanuthone D, scytalone/T3HN, and burnettramic acid A. Only one NRPS-like BGC was aligned to the recorded choline BGC. However, most predicted BGCs cannot be matched with well-characterized BGCs producing defined secondary metabolites (Table S3).

#### T1PKS BGC of melanin biosynthesis

**Fig. 5:**
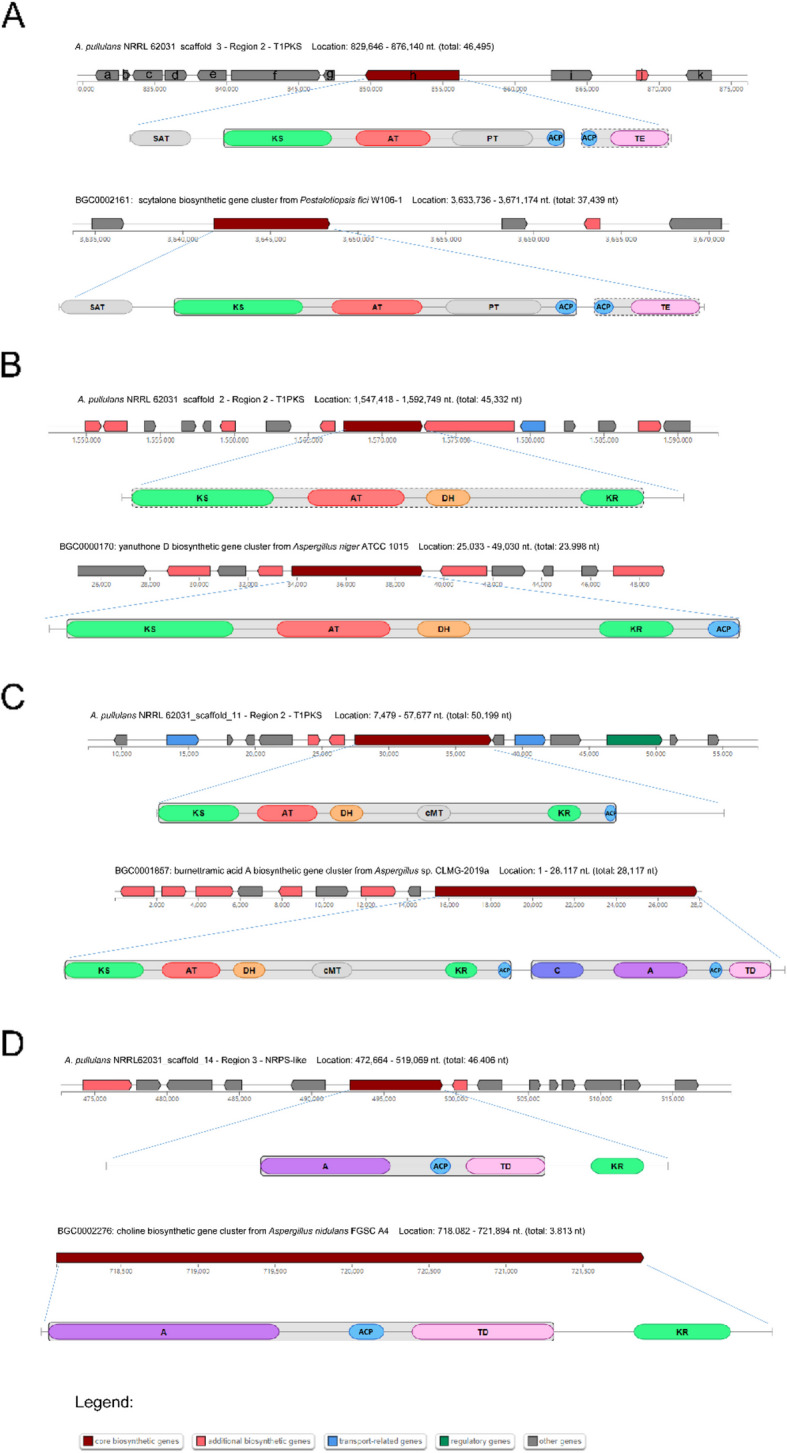
Predicted T1PKS and NRPS-like BGCs in *A. pullulans* NRRL 62031 and T1PKS BGC in *Pestalotiopsis fici* W106-1, *A. niger* ATCC 1015, *Aspergillus* sp. CLMG-2019a, and NRPS-like BGC in *Aspergillus nidulans* FGSC A4. The domain configurations of core biosynthetic genes are also shown. **A** The putative melanin T1PKS BGC in *A. pullulans* NRRL 62031 and the known scytalone T1PKS BGC in *Pestalotiopsis fici* W106-1, with domain configurations of their respective core biosynthetic genes. **B** The putative yanuthone D T1PKS BGC in *A. pullulans* NRRL 62031 and the known yanuthone D T1PKS BGC in *Aspergillus niger* ATCC 1015, with domain configurations of their respective core biosynthetic genes. **C** The putative burnettramic acid A T1PKS BGC in *A. pullulans* NRRL 62031 and the known burnettramic acid A T1PKS BGC in *Aspergillus* sp. CLMG-2019a, with domain configurations of their respective core biosynthetic genes. **D** The putative choline NRPS-like BGC in *A. pullulans* NRRL 62031 and the known choline NRPS-like BGC in *Aspergillus nidulans* FGSC A4, with domain configurations of their respective core biosynthetic genes. Abbreviations on domains of core biosynthetic genes: SAT, starter unit:ACP transacylase in aflatoxin biosynthesis; KS, ketosynthase domain; AT, acyltransferase domain; PT, product template domain; ACP, acyl-carrier protein domain; TE, thioesterase domain; DH, dehydratase domain; KR, ketoreductase domain; cMT, carbon methyltransferase; C, heterocyclization domain; TD, terminal reductase domain; A, adenylation domain

**Fig. 6:**
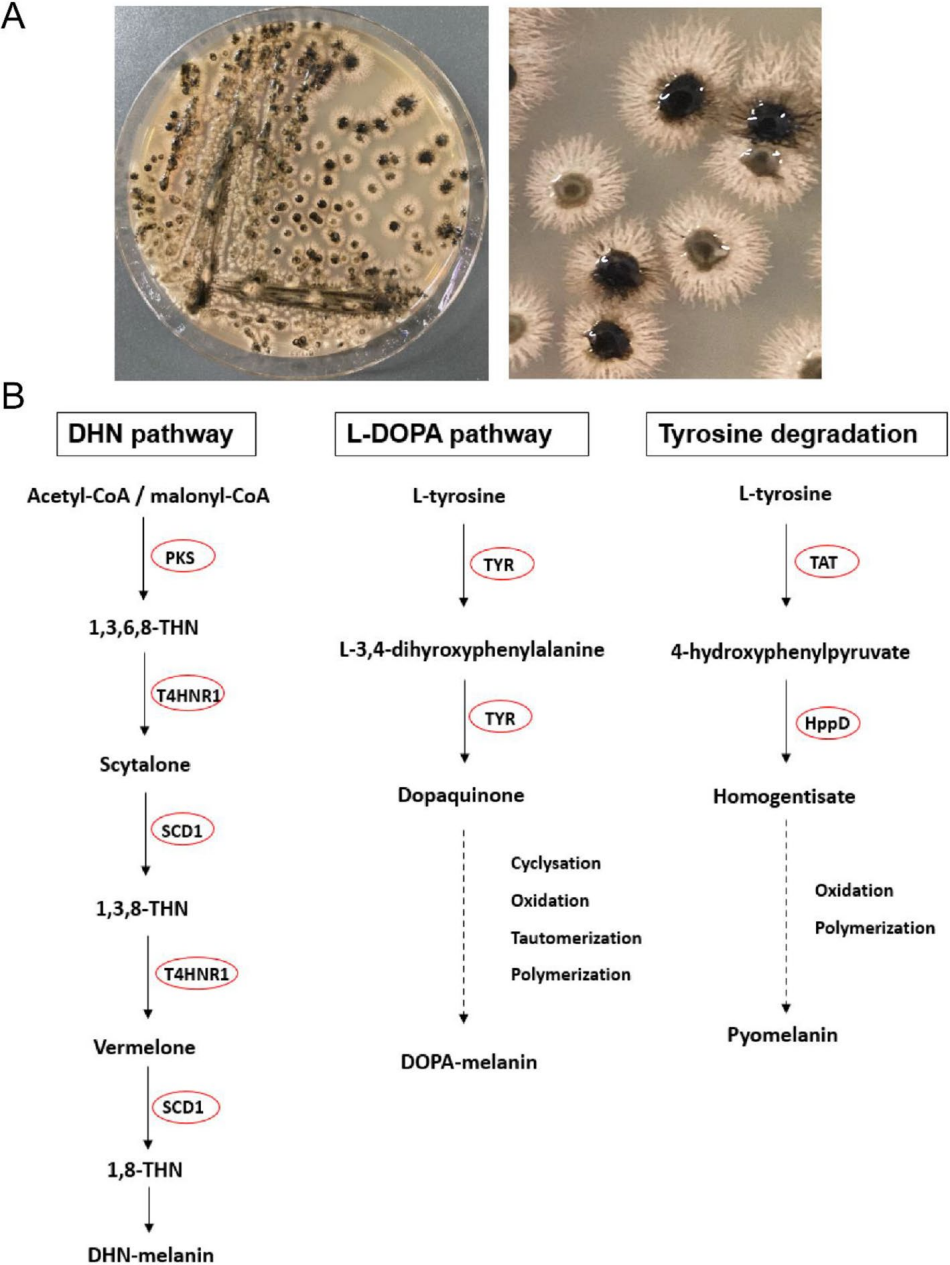
Melanin production as a trait of black yeasts. The observed colony pigmentation and the proposed melanin biosynthetic pathways of *A. pullulans* NRRL 62031. **A** The formation of melanin from *A. pullulans* NRRL 62031 on a malt agar plate. **B** The proposed biosynthetic pathways for DHN-melanin, DOPA-melanin, and pyomelanin synthesis in *A. pullulans* NRRL 62031. PKS, polyketide synthase; T4HNR1, tetrahydroxynaphthalene reductase; SCD1, scytalone dehydratase; TYR, tyrosinase; TAT, tyrosine aminotransferase; HppD, 4-hydroxyphenylpyruvate dioxygenase. The enzymes found in *A. pullulans* NRRL 62031 are circled in red

Melanin is regarded as an important black pigment for some potential applications (strong antioxidant, displaying free radical scavenging, anti-radiation, and anti-aging activity) [151].The T1PKS BGC on scaffold 3 of *A. pullulans* NRRL 62031 had a 40% similarity to the scytalone BGC in *Pestalotiopsis fici* W106-1*.* It was documented that scytalone is an important intermediate of DHN-melanin biosynthetic pathway [44]. Figure 5A shows that the core biosynthetic gene of the T1PKS BGC in *A. pullulans* NRRL 62031 had a completely identical arrangement of domains with that in *P. fici* W106-1, consisting of 1 SAT (starter unit: ACP transacylase) domain, 1 KS (ketosynthase) domain, 1 AT (acyltransferase) domain, 1 PT (product template) domain, 2 ACP (acyl-carrier protein) domains, and 1 TE (thioesterase) domain. In addition, eleven genes marked from a to b (Fig. 5A) on the T1PKS BGC (scaffold 3) of *A. pullulans* NRRL 62031 were further manually annotated through Blastp alignment to acquire their putative functions. It was predicted that four genes marked with a, b, e, and f encoded for uncharacterized proteins. The core biosynthetic gene (marked with h) was identified as a polyketide synthase with a similarity of 96.7% to the melanin-related polyketide synthase in *A. melanogenum* XJ5-1 (accession no. ALB35145.1). The gene (marked with i) was aligned to the putative transcription factor Cmr1 with a similarity of 97.7% (Table S4). It was shown that the transcription factor Cmr1 is crucial for promoting the expression of the *PKS1* gene and other genes contributing to melanin bioproduction in *A. melanogenum* XJ5-1 [151]. The gene (marked with j) was highly likely relevant to an ESC (*Elsinoë fawcetti*) reductase (XP_040884521.1) and a putative tetrahydroxynaphthalene (THN) reductase (XP_047768494.1) with an identity of 99.6% and 90.5%, respectively (Table S4). It has been well-documented that THN reductases take part in fungal DHN-melanin production [152, 153]. Other genes within the T1PKS BGC in *A. pullulans* NRRL 62031 were predicted to encode S-adenosyl-L-methionine-dependent methyltransferase, disulfide isomerase, JAB1/MPN domain-containing protein, and prefoldin (subunit 3) (Table S4). Intriguingly, it was found that the gene encoding a polyketide synthase (marked with h) and the gene encoding transcription factor Cmr1 (marked with i) in *A. pullulans* NRRL 62031 were adjacent with reversed transcription direction on the same BGC (Fig. 5A). It was in agreement with an evolution analysis that the *CMR1* and *PKS* genes have co-evolved in a contiguous “head-to-head” arrangement in most melanin-producing fungi [153]. All these results suggest that the T1PKS BGC on scaffold 3 in *A. pullulans* NRRL 62031 determined the production of DHN-melanin. In wet-lab experiments, it was observed that many colonies of *A. pullulans* NRRL 62031 became black when grown on a malt extract agar plate (Fig. 6A) and in the melanin production medium (data not shown), confirming melanin production.

#### Pullulan biosynthesis

**Fig. 7:**
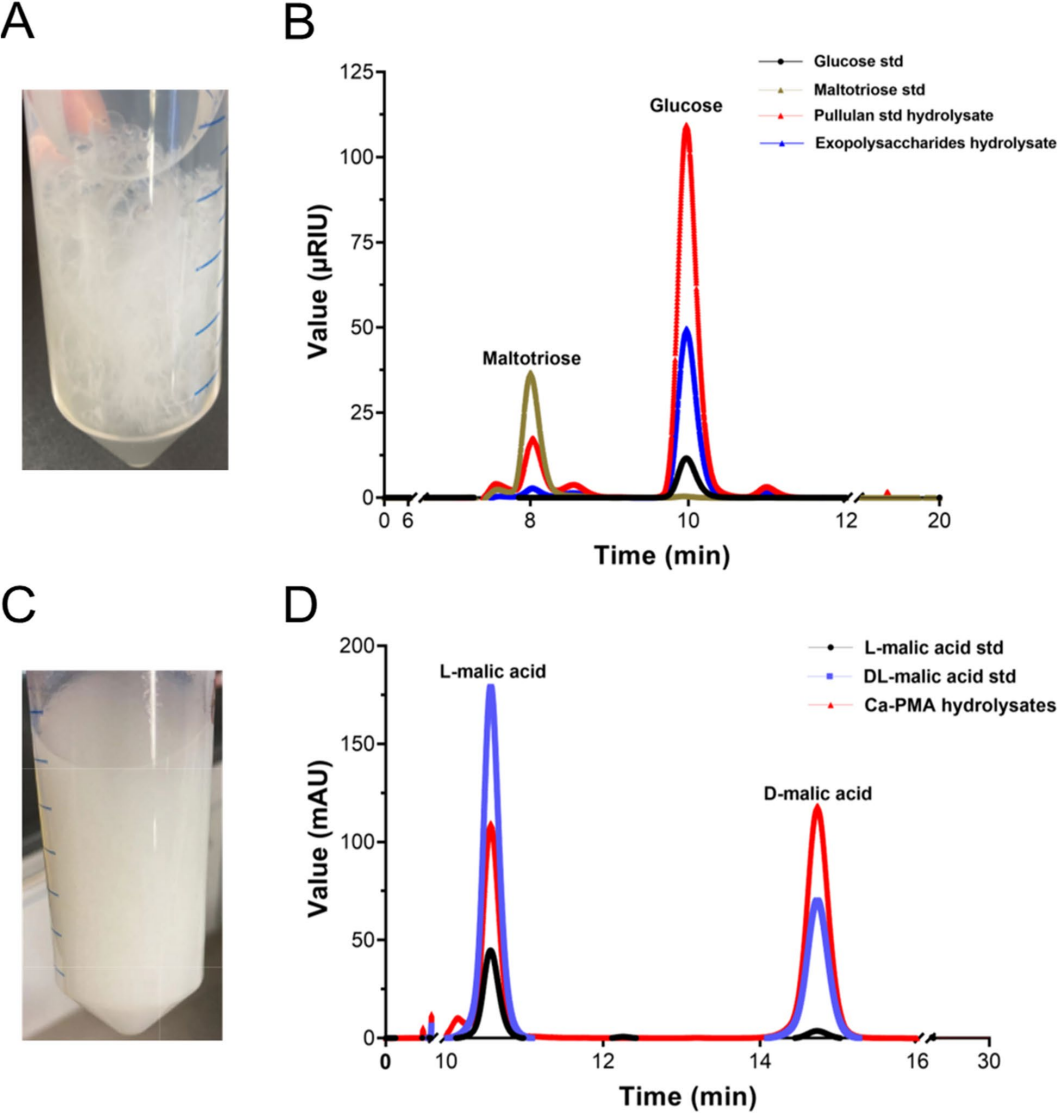
Non-dissolving forms of pullulan and Ca-PMA in ethanol and methanol, respectively, and the HPLC profiles of their hydrolysis products. **A** The flocculent form of pullulan precipitated in ethanol. **B** HPLC chromatograms of glucose, maltotriose, pullulan hydrolysate, and the hydrolysate of purified exopolysaccharides produced by *A. pullulans* NRRL 62031. **C** The powder-like form of Ca-PMA precipitated in methanol. **D** HPLC chromatograms of L-malic acid, a racemic mixture of L and D-malic acid, and the hydrolysate of the purified polymer secreted from *A. pullulans* NRRL 62031

Pullulan (poly-α−1,6-maltotriose), a trademark product from *Aureobasidium* spp., has been widely studied and is currently applied in the food, cosmetics, and pharmaceutical industries [27, 154]. However, no specific BGCs for producing pullulan or other exopolysaccharides are reported in literature. The pullulan synthetase encoded by the *PUL1* gene was found to be involved in pullulan synthesis in the siderophore-producing *A. pullulans* HN6.2 [155]. None of the putative pullulan synthetases in *A. pullulans* NRRL 62031 can be identified by aligning putative protein sequences to those from all public databases. However, a gene (g5656) product had an identity of 88.1% amino acid sequence aligned to the reported pullulan synthetase (accession number: AAQ05291.1) through manually running the Blastp search. Furthermore, it was demonstrated that a multidomain α-glucan synthase called AmAgs2 is the crucial enzyme for pullulan synthesis in *A. melanogenum* P16 [156–158]. Three genes (g5175, g6809, and g7000) coding for α-glucan synthases (XP_013428544.1, AYG85498.1, and AYG85496.1) in *A. pullulans* NRRL 62031 were assigned to the Nr database (Table S7). The flocculent exopolysaccharides immediately precipitated after 95% (v/v) cold ethanol was added to the supernatant of an *A. pullulans* NRRL 62031 culture broth (Fig. 7A). The HPLC results indicated that the enzymatic hydrolysates of the secreted exopolysaccharides resulted in a minor maltotriose peak (8.0 min) and a significant glucose peak (10.0 min). The hydrolysis products of the commercial pullulan also showed the same peaks (Fig. 7B). Taken together, these results suggest that *A. pullulans* NRRL 62031 can produce pullulan.

#### NRPS-like BGC of polymalate biosynthesis

PMA, a biopolymer of significant interest particularly in the biomedical field, was discovered to be produced by *Penicillium cyclopium* [159], *Physarum polycephalum* [160], and *Aureobasidium* spp. [40, 161]. It was identified that the protein product of the core biosynthetic gene on contig 6 within the NRPS-like BGC had a high similarity of 96.5% (Blastp) to that of the known polymalate (PMA) synthetase gene (QHD40385.1) in *A. melanogenum* ATCC 62921. In wet-lab experiments, powder-like white materials were precipitated after adding methanol to the supernatant from *A. pullulans* NRRL 62031 cultivated in the PMA production culture broth (Fig. 7C). Furthermore, the hydrolysis product peaks of the obtained powder-like white materials were identified as L-malic acid and D-malic acid (Fig. 7D). These in silico and HPLC analysis indicate that the NRPS-like BGC on contig 6 was responsible for PMA production in *A. pullulans* NRRL 62031.

#### T1PKS/NRPS-like BGC of polyol lipids biosynthesis

It was found that the protein product of the first core biosynthetic gene on contig 4 within the T1PKS/NRPS-like BGC shared 94.3% similarity with the reported polyketide synthase (AND82609.1) for polyol lipids biosynthesis in *A. melanogenum* 6–1–2. Furthermore, as displayed in Figure S4, the core biosynthetic gene was composed of one KS domain, one AT domain, one DH (dehydratase) domain, one ER (enoylreductase) domain, one KR (ketoreductase) domain, and one PKS-PP (phosphopantetheine acyl carrier protein group) domain. The reported polyol lipid-related PKS in *A. melanogenum* 6–1–2 has KS, AT, DH, ER, KR, and ACP (acyl-carrier protein domain) domains [162].

It has been reported that many strains of *Aureobasidium* spp. can produce extracellular polyol lipids, described as heavy oils, which have potential applications as novel biosurfactants [31, 163–165]. In a previous study, Saur et al. [34] identified and characterized the polyol lipids secreted from *A. pullulans* NRRL 62031.

#### Other T1PKS and NRPS-like BGCs predicted for the production of yanuthone D, burnettramic acid A, and choline

It was predicted that two T1PKS BGCs were relevant to producing yanuthone D and burnettramic acid A, which are antibiotic compounds. The T1PKS BGC on scaffold 2 shared 50% similarity with the known yanuthone D BGC from *Aspergillus niger* ATCC 1015. As displayed in Fig. 5B, the core biosynthetic genes related to yanuthone D production in *A. pullulans* NRRL 62031 and *A. niger* ATCC 1015 both contained the KS, the AT, the DH, and the KR domains. It should be noted that the yanuthone D BGC in *A. pullulans* NRRL 62031 had a gene for transportation. Yanuthone D displays a strong antimicrobial activity and is characterized as a meroterpenoid derived from the polyketide 6-methylsalicylic acid [166, 167].

The T1PKS BGC on scaffold 11 can be aligned to the known burnettramic acid A BGC from *Aspergillus* sp. CLMG-2019a with a similarity of 33%. As shown in Fig. 5C, the core biosynthetic genes for burnettramic acid A production in *A. pullulans* NRRL 62031 and *Aspergillus* sp. CLMG-2019a both contained six types of domains: the KS, AT, DH, cMT (carbon methyltransferase), KR, and ACP domains. However, more domains, including one C (heterocyclization) domain, one A (adenylation) domain, one TD (terminal reductase) domain, and one extra ACP domain were found in *Aspergillus* sp. CLMG-2019a. Additionally, one regulatory gene and two transporter-related genes with the same genetic orientation were present in the burnettramic acid A BGC of *A. pullulans* NRRL 62031. Burnettramic acid A displayed a comparable activity to amphotericin B against *Candida albicans* and it is a unique bolaamphiphilic scaffold composed of β-D-mannosyl residue linked to a pyrrolizidinedione unit by a 26-carbon alkyl chain [168, 169].

The NRPS-like BGC on scaffold 14 was aligned to the recorded choline BGC from *Aspergillus nidulans* FGSC A4 with an identity of 100%. The core biosynthetic gene on the NRPS-like BGC and the choline BGC had identical domain organization: A, ACP, TD, and KR domains (Fig. 5D). Moreover, two additional biosynthetic genes and eleven other genes were present on the NRPS-like BGC from *A. pullulans* NRRL 62031 but no other genes were found on that from *A. nidulans* FGSC A4. Choline is not only a necessary metabolite for the growth of filamentous fungi but also is recognized as an essential nutrient for humans [170, 171].

### Elucidation of biosynthetic pathways and transcriptional regulation by genome-mining

The protein sequences of the encoded functional genes assigned to GO, KEGG, NCBI-Nr, and ATFDB public databases via the BLAST (v2.2.31) with an E-value below 1e^−5^ were used to predict if *A. pullulans* NRRL 62031 has the complete pathways for the biosynthesis of pullulan, polymalate, and polyol lipids.

#### Melanin

To date, it has been well-documented that there are three melanogenic pathways, including the DHN pathway, L-DOPA pathway, and tyrosine degradation [153, 172, 173]. As shown in Fig. 6B, the polyketide synthase (PKS) converts acetyl-CoA or malonyl-CoA precursor into 1,3,6,8-tetrahydroxynaphthalene (1,3,6,8-THN), followed by a series of reduction and dehydration to THN-melanin under the catalysis of tetrahydroxynaphthalene reductase (T4HNR1) and scytalone dehydratase (SCD1) [174]. DOPA-melanin synthesis is mainly catalyzed by tyrosinase (TYR) from L-tyrosine [175]. Tyrosine aminotransferase (TAT) and 4-hydroxyphenylpyruvate dioxygenase (HppD) are two key enzymes for pyomelanin production [176]. Moreover, it has been confirmed that the cell wall integrity (CWI) signaling pathway affects the activity of the transcriptional activator Swi4, which can upregulate the expression of the *CMR1* gene [151].

As analyzed above, the core biosynthetic gene (g2262) of T1PKS BGC on scaffold 3 encodes a polyketide synthase with a high sequence identity to the reported melanin-related polyketide synthase in *A. melanogenum* XJ5-1. One gene (g7053) coded for a scytalone dehydratase (KEQ60876.1), two genes (g2264 and g5807) coded for tetrahydroxynaphthalene reductases, six genes (g941, g1154, g3541, g5277, g7018, and g8919) coded for tyrosinases, one gene (KEQ63012.1) coded for a tyrosine aminotransferase, four genes (g783, g4074, g4754, and g6112) coded for 4-hydroxyphenylpyruvate dioxygenases, and one gene (g2263) coded for the transcription factor Cmr1 (XP_013431775.1) (Table S5). However, the transcription factor Swi4 (KEQ58325.1) with one copy can only be aligned to the ATFDB (*Aureobasidium* transcription factor database) pipeline (Table S6). Moreover, one transcription factor Cmr1 can also be aligned to the ATFDB database (Table S6), which was the same as the transcription factor Cmr1 (XP_013431775.1) predicted based on the Nr database (Table S5).

Therefore, *A. pullulans* NRRL 62031 is proposed to produce three types of black pigments, with DHN-melanin synthesis being regulated by the CWI signaling pathway through the transcription factors Swi4 and Cmr1.

#### Pullulan

To date, several key enzymes and their encoding genes for pullulan biosynthesis have been identified. As shown in Figure S5, the pullulan precursor UDP-glucose is formed from 1-phosphate-glucose and UTP (uridine triphosphate) under the catalysis of uridine diphosphoglucose pyrophosphorylase (UGP). 1-phosphate-glucose is derived from 6-phosphate-glucose through the catalysis of α-phosphoglucose mutase, and 6-phosphate-glucose is a significant intermediate of glycolysis [177, 178]. At first, it was proposed that UDP-glucose is converted into the glucose-containing lipid intermediate (Lph-glucose) via the catalysis of glucosyltransferase. The Lph-glucose is then polymerized into pullulan by a series of glucosyl-transfer reactions [177]. It has been detected that not only the activity of α-phosphoglucose mutase and UDPG-pyrophosphorylase but also that of glucosyltransferase were high in *A. pullulans* Y68 featuring a high yield of pullulan [178]. It was found that two genes (g852 and g7458) coded for α-phosphoglucose mutase (KEQ59353.1 and XP_007780174.1), and one gene (g4829) coded for UDPG-pyrophosphorylase (XP_023890373.1) (Table S7). Moreover, according to the CAZy database, a total of 285 enzymes (GT class) in *A. pullulans* NRRL 62031 were assigned to the glycosyltransferases (GTs) class, constituting the second largest proportion (30%) of all predicted carbohydrate-active enzymes (Fig. 4C). This may suggest that the activities of enzymes involved in transferring glucosyl groups in *A. pullulans* NRRL 62031 were exceedingly high. It has been reported that the *UGT1* gene encoding a UDP-glucose:glycoprotein glucosyltransferase-like protein is involved in pullulan production in *A. melanogenum* P16 [179]. Until now, this is the only well-characterized glucosyltransferase-related gene in *Aureobasidium* spp. for pullulan synthesis. Therefore, it is necessary to identify other genes coding for key glucosyltransferases for pullulan biosynthesis in the future. One gene (g2658) coding for UDP-glucose:glycoprotein glucosyltransferase (AQQ13387.1) was present in *A. pullulans* NRRL 62031 (Table S7).

Chen et al. [156] clarified that the glycogenins (Glg1 and Glg2), sterol glucosyltransferase (Sgt1), and ceramide β-glucosyltransferase (Gcs1) can contribute to the synthesis of short-chain α−1,4-glucans (primers of pullulan) derived from UDP-glucose. After the formation of pullulan primers, their length is extended by the intracellular glycogen synthetase domain (Gys_D) of a multidomain α-glucan synthase called AmAgs2 in *A. melanogenum* P16, resulting in a long chain of α−1,4-glucans (precursors of pullulan). The exopolysaccharide transport domain (EPST_D) of the AmAgs2 can transport the long chain of α−1,4-glucans to the periplasmic space. Then, the extracellular α-amylase catalytic domain of the AmAgs2 (Amy_D) hydrolyzes the endo-α−1,4-linkages of α−1,4-glucans to form maltotriose repeats. Finally, the maltotriose repeats are anchored to the Lph-glucose and polymerized into pullulan [156]. One gene (g2104) coding for a sterol glucosyltransferase (KEQ62498.1), three genes (g5175, g6809, and g7000) coding for α-glucan synthases (XP_013428544.1, AYG85498.1, and AYG85496.1) assigned to the Nr database and one gene (g4364) coding for ceramide β-glucosyltransferase, and two genes (g840 and g7961) coding for glycogenins aligned to the KEGG database were found in *A. pullulan* NRRL 62031. Intriguingly, one gene (g8879) encoding a glycogen synthase and five genes (g1316, g3298, g3502, g6635, and g8572) encoding α-amylases were also found (Table S7). It can be speculated that not only multidomain α-glucan synthases on the cell membrane but also free glycogen synthases and α-amylases might be engaged in the formation of the long chain α−1,4-glucans and the release of maltotriose units in *A. pullulans* NRRL 62031.

The transcription factor Msn2 (XP_013341874.1) in *A. pullulans* NRRL 62031 was aligned to that from *A. subglaciale* EXF-2481 in the ATFDB pipeline (Table S6). The regulator Msn2, a general stress response regulator, positively regulates the expression of the *UGP1* gene, which encodes UDPG-pyrophosphorylase responsible for UDP-glucose formation, taking part in pullulan biosynthesis. The subcellular localization of the Msn2 is only controlled by cAMP-PKA signaling pathway [157, 180–182]. The regulator CreA, a global zinc finger regulator, can downregulate the genes for pullulan biosynthesis [157, 183]. Additionally, it was demonstrated that the pH transcription factor Appacc (PacC in *A. pullulans*) could up-regulate the production of pullulan and alter the cell growth in *A. pullulans* [184]. The GATA-type transcriptional factors, including transcriptional activator AreA and transcriptional repressor AreB of the nitrogen catabolite repression (NCR) system, could control pullulan biosynthesis in *A. melanogenum* P16 [180]. A putative gene (g274) coding for the regulator CreA (AIZ77451.1) aligned to the Nr database with one copy (Table S7). The transcription factors AreA (AWD76385.1) and AreB (AWD76386.1) were aligned to the Nr database (Table S7). However, the genes encoding CreA, AreA, and AreB were not aligned to the ATFDB database. A regulator PacC (KEQ83946.1) was present in *A. pullulans* NRRL 62031 (Table S6).

Taken together, these results suggest that *A. pullulans* NRRL 62031 possessed all genes coding for important enzymes implicated in pullulan synthesis, and the synthesis could be controlled by Msn2, CreA, AreA, AreB, and PacC.

#### Polymalate

Recently, the mechanism of polymalate polymerization has been proposed, and the key enzyme for the polymerization of malate into PMA has been discovered [185]. Specifically, a non-ribosomal peptide synthetase (NRPS) containing an A-like domain (adenylation), a T-domain (thiolation), and a C-like domain (condensation) was a transmembrane enzyme for PMA synthesis in *A. melanogenum* ATCC 62921, and it was the first enzyme identified for PMA polymerization in *Aureobasidium* spp. [185]. As described above, the core biosynthetic gene (g3625) on contig 6 within the NRPS-like BGC had a high similarity of 96.5% (Blastp) to that of the known polymalate (PMA) synthetase gene (QHD40385.1) in *A. melanogenum* ATCC 62921 by manual Blastp alignment.

As shown in Figure S6, it has been proposed that three pathways contribute to the formation of intracellular malate, the only precursor for PMA synthesis, including the oxidative TCA cycle, the cytosolic reductive pathway, and the glyoxylate shunt [186, 187]. In the TCA cycle, malate is a crucial intermediate converted from fumaric acid under the catalysis of fumarase (FUM). In the glyoxylate cycle, glyoxylate and acetyl-CoA are condensed to produce malate under the catalysis of malate synthase (MSE). In the reductive pathway, oxaloacetate plays a significant role. First, it is carboxylated from pyruvate under the catalysis of pyruvate carboxylase (PYC) accompanied by the fixation of one CO_2_ molecule. Then, it is reduced to malate catalyzed by cytosolic malate dehydrogenase (cyMDH) [186]. Two genes (g6753 and g7477) coding for fumarases, one gene (g7480) coding for malate synthase (OCL10598.1), two genes (g667 and g6960) coding for pyruvate carboxylases, and four genes (g2315, g2655, g4110, and g5765) coding for malate dehydrogenases can be assigned to the Nr or KEGG databases (Table S8). It was known that malate dehydrogenases are encoded by three genes in traditional yeast. One of these enzymes is localized in the cytoplasm, whereas the other two enzymes mediate the dehydrogenation in mitochondria and peroxisomes [188]. Four putative malate dehydrogenases need to be further classified and studied in *A. pullulans* NRRL 62031.

It was documented that the activator Crz1 from the Ca^2+^-signaling pathway can upregulate the expression level of PMA synthetase, thereby leading to higher polymalate production [185, 186]. The Crz1 can be discovered in most genomes of *Aureobasidium* spp. [78]. It was also found in the genome of *A. pullulans* NRRL 62031 based on a sequence from the ATFDB database (Table S6). The enzyme PPTase (phosphopantetheinyl transferase) can induce the T domain in the PMA synthetase through phosphopantetheine (PP) addition, and the dephosphorylated form of the Crz1 mediated by the Ca^2+^-signaling pathway leads to its translocation from the cytoplasm to the nucleus, inducing the expression of several target genes including the PMA synthetase gene [185, 186]. One gene (g2273) coding for the enzyme PPTase (AST22499.1) was present in *A. pullulans* NRRL 62031 (Table S8). Furthermore, the transcription activator AreA, as one of the key components in the nitrogen catabolite repression (NCR) system, was proven to regulate nitrogen assimilation and biosynthesis of PMA [189]. However, the regulation evidence of AreB, the transcription repressor of the NCR system, has not been elucidated in PMA biosynthesis from *Aureobasidium* strains. The transcription factors AreA and AreB were found in *A. pullulans* NRRL 62031 (Table S7). It has also been reported that the GATA-type transcription factor NsdD can positively regulate PMA biosynthesis in *A. melanogenum* ATCC 62921 [186]. The transcription factor NsdD (AXS67923.1) was found in *A. pullulans* NRRL 62031 (Table S8).

The results suggest that *A. pullulans* NRRL 62031 contained all the genes coding for crucial enzymes for intracellular malate synthesis and PMA condensation. Moreover, this strain may include the calcium ion-responsive transcription factor Crz1, the regulators of the NCR system AreA and AreB, and the global regulator NsdD, which might regulate PMA production.

#### Polyol lipids

The proposed biosynthetic pathway for polyol lipid synthesis is exhibited in Figure S7. The esterase encoded by the esterase gene (*EST1*) is a key enzyme for polyol lipid biosynthesis in *A. melanogenum* 6–1–2 [43, 162]. Particularly, the esterase takes part in the formation of the ester bond between 3,5-dihydroxydecanoic acid and a single polyol [47, 190]. A gene (g2600) coding for an esterase (AYC07631.1) was found in *A. pullulans* NRRL 62031 (Table S9). Notably, genes encoding other types of esterases were also present in *A. pullulans* NRRL 62031, such as carboxylesterases, feruloyl esterases, acetyl xylan esterases, pectinesterases (data not shown). The key enzymes responsible for the synthesis of mannitol and arabitol are mannitol 1-phosphate-5-dehydrogenase (MPDH) and mannitol dehydrogenase (MtDH), and arabitol dehydrogenase (ArDH), respectively [34, 43]. The enzyme MtDH (AST36438.1) and ArDH (AYC07633.1) aligned to the Nr database had one copy each in *A. pullulans* NRRL 62031, whereas one copy of the gene *MPDH* (g284) was found aligned to the KEGG database (Table S9). Some enzymes involved in forming other headgroups, such as glycerol, xylitol, galactitol, sorbitol, or threitol, are necessary to be discovered in *A. pullulans* NRRL 62031 as polyol lipids with varied head groups have different bioactivities [43]. The 3,5-dihydroxydecanoic acid, the tail group of the molecular structure of polyol lipids, is formed by the continuous condensation of acetyl-CoA and malonyl-CoA under the catalysis of the highly reducing polyketide synthase (HR-PKS) [162]. The putative polyketide synthase that contributes to 3,5-dihydroxydecanoic acid (AND82609.1) was discovered in the genomic DNA of *A. pullulans* NRRL 62031 (Table S9). Additionally, this gene was the first core biosynthetic gene on contig 4 within the T1PKS/NRPS-like BGC, and it showed a high similarity to the known polyol lipid biosynthesis-related polyketide synthase as described above.

Furthermore, it was shown that the ACP domain in the HR-PKS can be post-translationally phosphopantetheinylated by a phosphopantetheinyl transferase (PPTase), and this enzyme could regulate the HR-PKS activity, thus affecting the polyol lipid production in *A. melanogenum* 6–1–2 [162]. One gene (g2273) encoding a PPTase (AST22499.1) was present in *A. pullulans* NRRL 62031 (Table S8). It was also demonstrated that the *PKS1* and *EST1* genes can be strongly up-regulated by the zinc finger transcriptional activator Gal1 encoded by the *GAL1* gene in *A. melanogenum* 6–1–2 [162]. However, the activator Gal1 was not found in *A. pullulans* NRRL 62031. Polyol lipid production in *A. melanogenum* 6–1–2 has been shown to be regulated by the HOG1 and cAMP-PKA signaling pathways via the global transcriptional activator Msn2 in *A. melanogenum* 6–1–2 [191]. As stated above, Msn2 is also a key regulator to affect pullulan production. Only one homolog to transcription factor Msn2 (XP_013341874.1) was present in *A. pullulans* NRRL 62031 (Table S6).

Taken together, *A. pullulans* NRRL 62031 contained putative genes coding for key enzymes including a mannitol 1-phosphate-5-dehydrogenase, a mannitol dehydrogenase, an arabitol dehydrogenase, a highly reducing polyketide synthase, an esterase, and a phosphopantetheinyl transferase which are likely responsible for polyol lipid production and a transcription factor Msn2 for the regulation of polyol lipid production.

#### Other possible value-added metabolites

*A. pullulans* NRRL 62031 might synthesize other valuable products such as fructooligosaccharides, gluconic acid, β−1,3-glucan, and aureobasidin A. It was found that four genes (g1443, g3017, g7938, and g7939) coding for β-fructofuranosidases were present in *A. pullulans* NRRL 62031 (Table S10). β-fructofuranosidases (invertases) are responsible for producing fructooligosaccharides from sucrose by transfructosylation [192]. Fructooligosaccharides are widely applied as prebiotics, which are beneficial for human health by stimulating the gut microbiota balance [193]. Two genes (g1252 and g1296) coding for glucose oxidases were also present in *A. pullulans* NRRL 62031 (Table S10). Glucose oxidases can convert glucose into gluconic acid [194, 195]. In addition, only one gene (g2024) encoding a β−1,3-glucan synthase was found in *A. pullulans* NRRL 62031 (Table S10). β−1,3-glucan synthase is likely relevant to β−1,3–1,6-glucan biosynthesis. It has been reported that β−1,3–1,6-glucan can be applied as a functional food ingredient with health benefits [196]. In addition, the gene (g8358) product can be aligned to the known aureobasidin A-producing enzyme complex (ACJ04424.1) with an identity of 22.4% through manual Blastp. This enzyme was discovered in *A. pullulans* BP-1938 and aureobasidin A is a cyclic nonadepsipeptide showing strong fungicidal activity [197].

Taken together, the presence of putative genes encoding β-fructofuranosidases, glucose oxidases, a β−1,3-glucan synthase, and an aureobasidin A-producing enzyme complex in *A. pullulans* NRRL 62031 suggests that *A. pullulans* NRRL 62031 has the potential to produce fructooligosaccharides, gluconic acid, β-glucan, and aureobasidin A. These compounds of commercial interest are awaiting experimental validation, which would expand the biomanufacturing scope of this strain.

## Conclusion

In the present study, the genome and phenotype of *A. pullulans* NRRL 62031, sampled from a leaf in Thailand, were investigated. The phylogenetic analysis and average nucleotide identity analysis suggested that *A. pullulans* NRRL 62031 should be reclassified as a strain of *A. melanogenum*. KEGG, GO, KOG, and CAZy annotation accompanied by wet-lab experiments demonstrated that *A. pullulans* NRRL 62031 possessed a considerable number of genes involved in a variety of metabolic processes and was able to utilize many carbon substrates (saccharides, polyols, and organic acids) and hydrolyze biopolymers (cellulose, hemicellulose, pectin, and starch). The antiSMASH analysis indicated the presence of T1PKS for the synthesis of scytalone, yanuthone D, and burnettramic acid A, and a NRPS-like BGC for choline synthesis. The genome mining data and wet-lab experiments demonstrated that *A. pullulans* NRRL 62031 could produce melanin, pullulan, polymalate, and polyol lipids. These molecules may be interesting for application in pharmaceuticals, cosmetics, and food. In conclusion, this study provides valuable insights into the potential of *A. pullulans* NRRL 62031 for biomanufacturing applications, including the production of various bioproducts.

## Supplementary Information

Supplementary Material 1.

## Data Availability

The whole-genome shotgun project has been deposited at DDBJ/ENA/GenBank under the accession no. JALBUZ000000000. The version described in this paper is version no. JALBUZ010000000. The respective raw sequencing data have been deposited at the Sequence Read Archive under the accession number no. SRR17771678.
