# Supplementary material for: Genome mining the black-yeast Aureobasidium pullulans NRRL 62031 for biotechnological traits
Source: BMC Genomics. 2025 Mar 13;26:244. doi: 10.1186/s12864-025-11395-2 (PMC11905612; doi:10.1186/s12864-025-11395-2)
Supplement: Supplementary file 1 — Supplementary Material 1. [file 12864_2025_11395_MOESM1_ESM.pdf]

Supplementary information

Genome mining the black-yeast *Aureobasidium pullulans* NRRL 62031 for biotechnological traits

Difan Xiao[1], Marielle Driller[1], Karla Stein[1], Lars M. Blank[1], Till Tiso[1]*

Affiliations

[1] Institute of Applied Microbiology – iAMB, Aachen Biology and Biotechnology – ABBt, RWTH Aachen University, Aachen, Germany

*Corresponding author:

Till Tiso, till.tiso@rwth-aachen.de

iAMB - Institute of Applied Microbiology, ABBt - Aachen Biology and Biotechnology, Worringer Weg 1, RWTH Aachen University, 52074 Aachen, Germany

Supplementary Figures

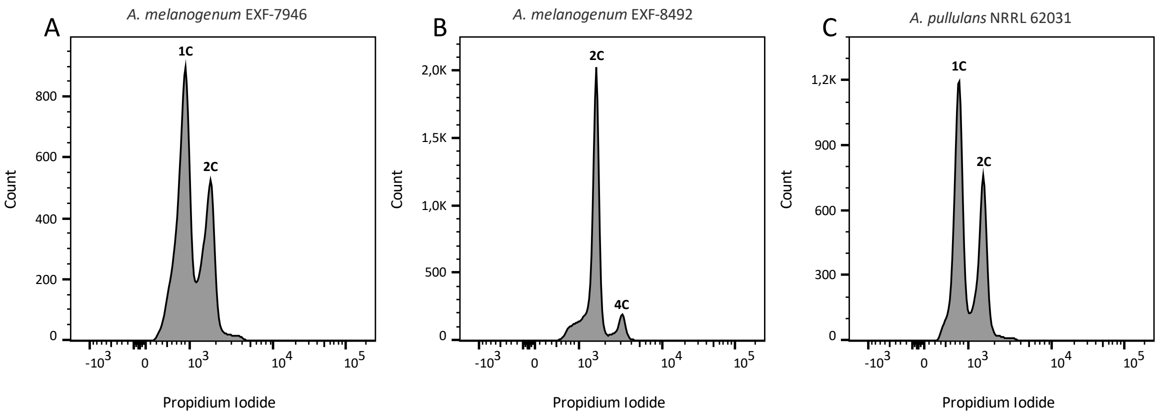

Figure S1. Propidium iodide fluorescence of stained DNA for *Aureobasidium* strains. The histogram shows two fluorescent peaks for each strain, with the first one showing the fluorescence of cells in the G1 phase of the cell cycle and the second one showing the fluorescence of cells in the G2 phase. By comparing the fluorescent intensity of the haploid (A) and diploid (B) reference strains, strain *A. pullulans* NRRL 62031 (C) was confirmed to have a haploid genome.

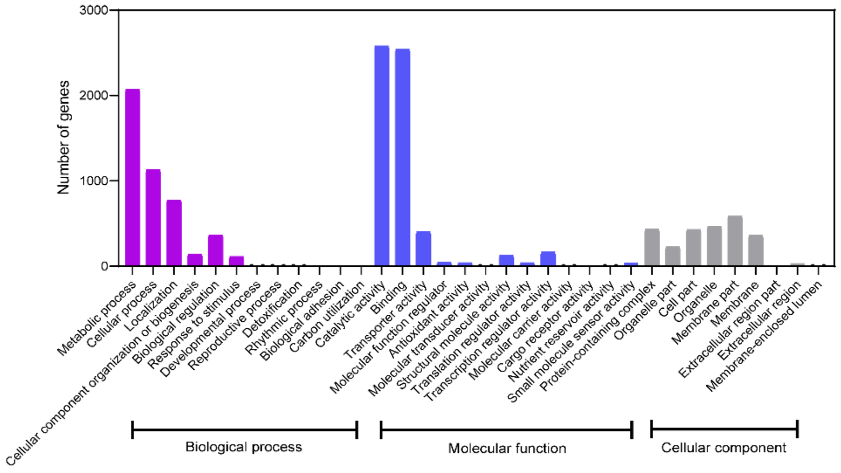

Figure S2. Gene ontology (GO) classification of the proteins deduced from the genome of *A. pullulans* NRRL 62031.

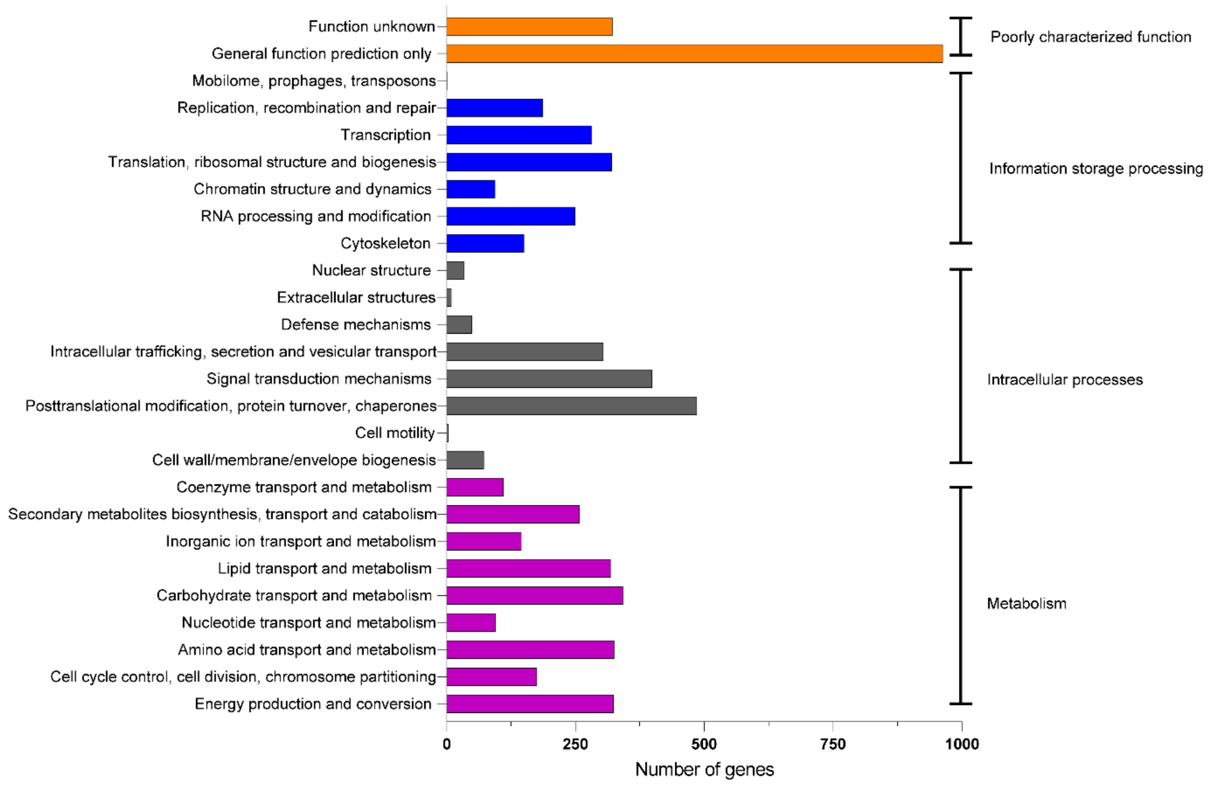

Figure S3. Eukaryotic orthologous groups (KOG) function classification of the proteins deduced from the genome of *A. pullulans* NRRL 62031.

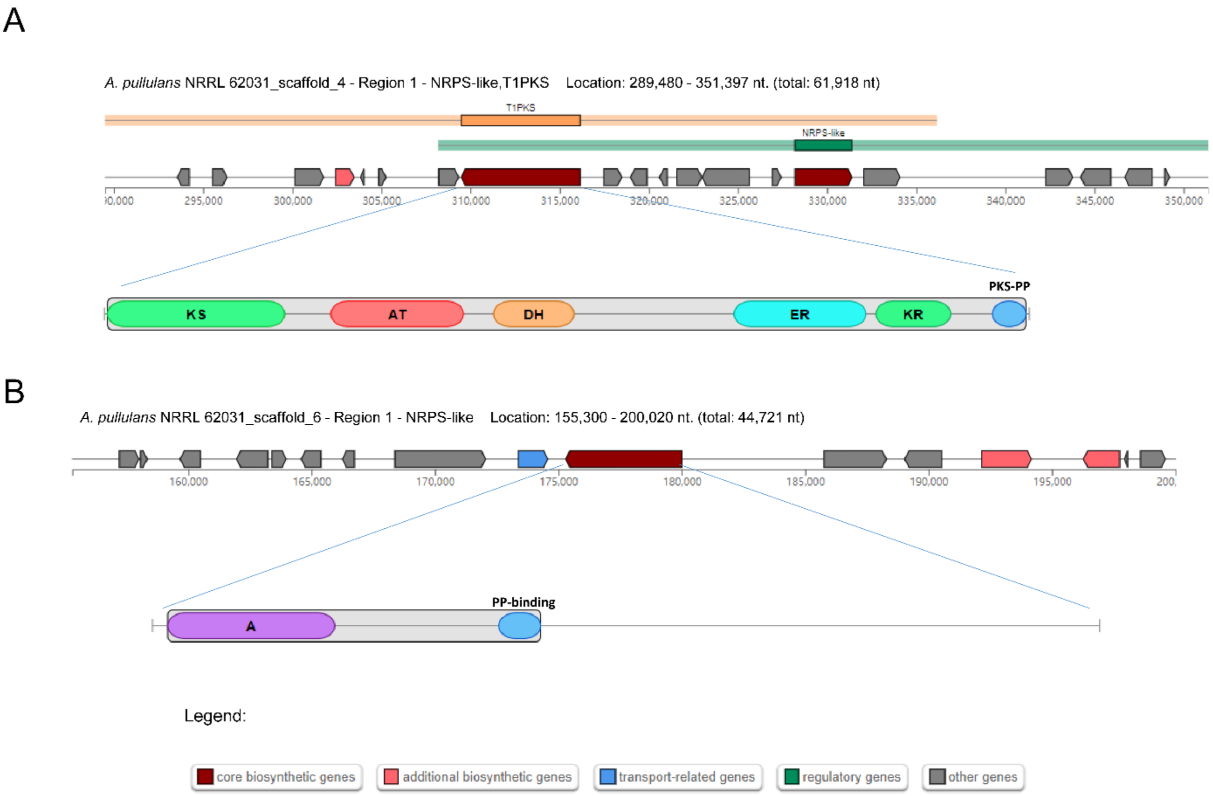

Figure S4. T1PKS/NRPS-like biosynthetic gene cluster (BGC) for polyol lipid production and NRPS-like BGC for polymalate production in *A. pullulans* NRRL 62031 and domain configuration of the core biosynthetic gene. (A) Polyol lipids T1PKS/NRPS-like BGC and domain configuration in the core biosynthetic gene. (B) Polymalate NRPS-like BGC and domain configuration in the core biosynthetic gene. Abbreviations on domains of core biothethetic genes: SAT, starter unit:ACP transacylase in aflatoxin biosynthesis; KS, ketosynthase domain; AT, acyltransferase domain; DH, dehydratase domain; KR, ketoreductase domain; A, adeylation domain; ER, enoylreductase domain; PKS-PP, phosphopantetheine acyl carrier protein group; PP-binding, phosphopantetheine-binding.

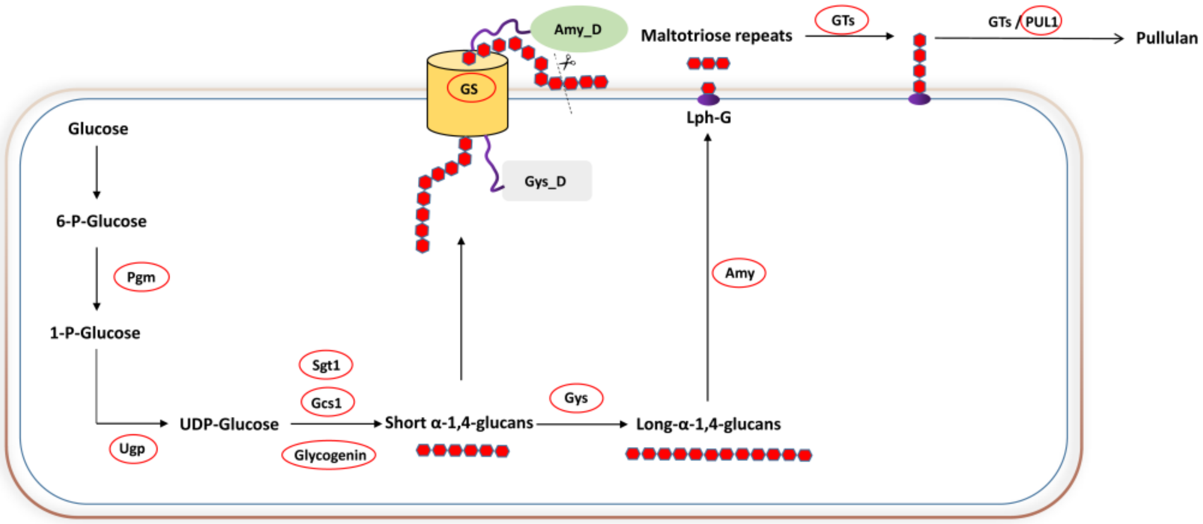

Figure S5. The proposed biosynthetic pathways for pullulan production in *A. pullulans* NRRL 62031. PGM, phosphoglucomutase; Ugp, UTP-glucose-1-phosphate uridylyltransferase; Sgt1, sterol glucosyltransferase; Gcs1, ceramide β-glucosyltransferase; Gys, glycogen synthase; Amy, α-amylase; GS, α-glucan synthase; Lph-G, Lph-glucose; GTs, glucosyltransferases; PUL1, pullulan synthetase. The enzymes found in *A. pullulans* NRRL 62031 are circled in red.

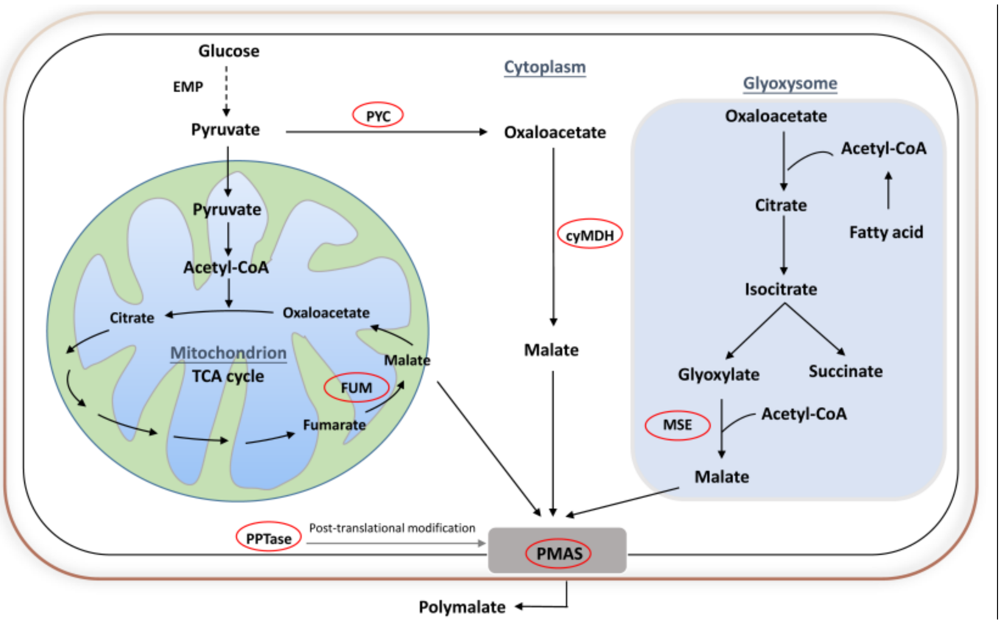

Figure S6. The proposed biosynthetic pathways of polymalate in *A. pullulans* NRRL 62031. FUM, fumarase; PYC, pyruvate carboxylase; cyMDH, cytosolic malate dehydrogenase; MSE, malate synthase; PMAS, polymalate synthetase; PPTase, phosphopantetheinyl transferase. The enzymes found in *A. pullulans* NRRL 62031 are circled in red.

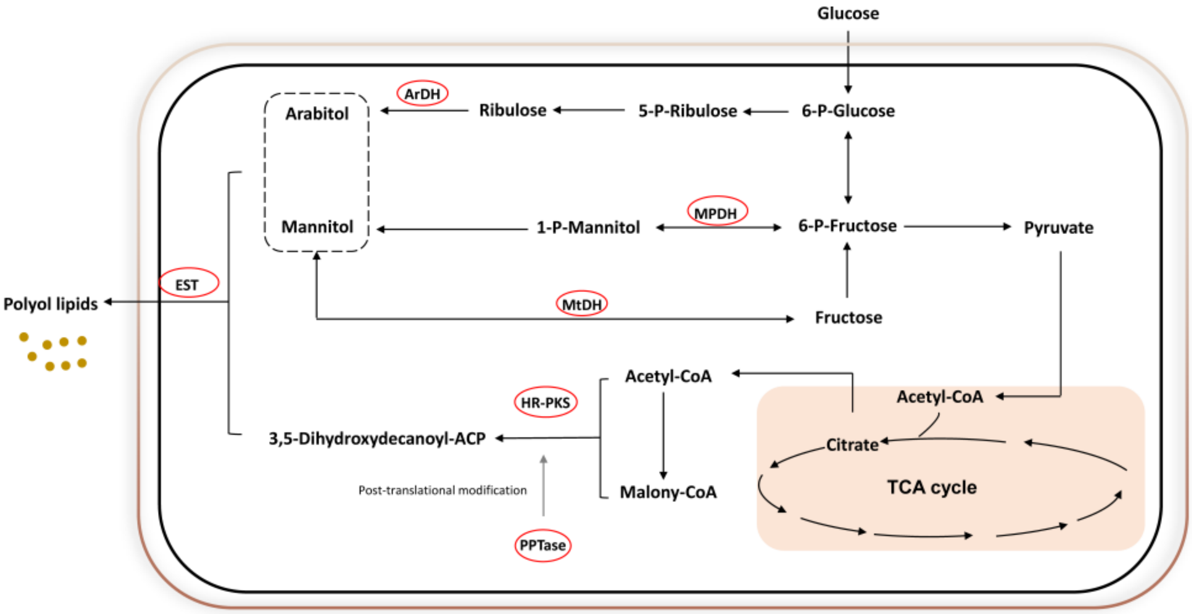

Figure S7. The proposed biosynthetic pathways for polyol lipid synthesis in *A. pullulans* NRRL 62031. ArDH, arabitol dehydrogenase; MPDH, mannitol-1-phosphate dehydrogenase; PKS, polyketide synthase; PPTase, phosphopantetheinyl transferase; EST, esterase. The enzymes found in *A. pullulans* NRRL 62031 are circled in red.

Supplementary Tables

Table S1. The general information of 16 publicly available *Aureobasidium* spp.

Organism	Strain	Isolation source	Reference
Aureobasidium pullulans	EXF-150	Slovenia, hypersaline saltern water	Gostinčar *et al.*, 2014
Aureobasidium pullulans	EXF-8828	Argentina, glacial meltwater	Gostinčar *et al.*, 2019
Aureobasidium pullulans	EXF-3780	Puerto Rico, microbial mat, bottom of the sea water	Gostinčar *et al.*, 2019
Aureobasidium pullulans	EXF-11900	Croatia, kitchen refrigerator rubber seal	Gostinčar *et al.*, 2019
Aureobasidium pullulans	EXF-9399	Greece, black olives fermentation	Gostinčar *et al.*, 2019
Aureobasidium pullulans	EXF-3380	Germany, oak slime flux	Gostinčar *et al.*, 2019
Aureobasidium melanogenum	CBS 110374	Thailand, public fountain	Gostinčar *et al.*, 2014
Aureobasidium melanogenum	EXF-3233	Japan, deep sea	Cernosa *et al.*, 2021
Aureobasidium melanogenum	EXF-926	Norway, surface glacial ice	Cernosa *et al.*, 2021
Aureobasidium melanogenum	EXF-5590	Slovenia, rubber seal dishwasher	Cernosa *et al.*, 2021
Aureobasidium melanogenum	EXF-10726	Brazil, integument of a male alate ant	Cernosa *et al.*, 2021
Aureobasidium namibiae	CBS 147.97	Namibia, dolomitic desert marble	Gostinčar *et al.*, 2014
Aureobasidium subglaciale	EXF-2481	Norway, glacial ice	Gostinčar *et al.*, 2014
Aureobasidium uvarum	AWR4620	Australia, shiraz grape juice	Onetto *et al.*, 2020
Aureobasidium vineae	AWR4619	Australia, shiraz grape juice	Onetto *et al.*, 2020
Aureobasidium mustum	AWR4233	Australia, shiraz grape juice	Onetto *et al.*, 2020

Table S2. Statistics of repeats in the genome of *A. pullulans* NRRL 62031

DNA repeat sequence description	Number
Short interspersed nuclear elements	0
Long interspersed nuclear elements	0
Long terminal repeats	0
DNA transposons	0
Unclassified interspersed repeats	271
Small RNA	54
Satellite RNA	0
Simple repeats	2615
Sequences with low complexity	387

Table S3. The identified biosynthetic gene clusters through antiSMASH Fungal 7.1.0 version.

Region	Type	Range	Most similar known cluster	Similarity
1.1	fungal-RiPP-like	781,683-842,613		
3.1	terpene	805,379-827,314		
3.2	T1PKS	1,547,418-1,592,749	yanuthone D (polyketide)	50%
4.1	T3PKS	31,610-74,499		
4.2	T1PKS	829,646-876,140	scytalone/T3HN (polyketide)	40%
5.1	T1PKS, NRPS-like	289,480-351,397		
6.1	NRPS-like	242,572-285,697		
7.1	NRPS-like	155,300-200,020		
7.2	terpene	654,985-676,912		

11.1	NRPS-like	617,162-660,734		
12.1	T1PKS	7,479-57,677	burnettramic acid A (Alkaloid+NRP+Polyketide:Iterative type I polyketide)	33%
14.1	betalactone	387,380-417,848		
15.1	NRPS, betalactone	137,848-183,969		
15.2	NRPS-like	331,265-373,787		
15.3	NRPS-like	472,664-519,069	Choline (NRP)	100%
17.1	T1PKS, NRPS-like, indole	160,360-236,178		
18.1	NAPAA	58,321-93,368		
19.1	terpene	353,104-377,282		
25.1	T1PKS1	1-33,887		
25.2	NRP-metallophore, NRPS	120,920-183,890		
26.1	fungal-RiPP-like	67,109-130,298		
38.1	terpene	14,651-36,587		
45.1	Terpene1	1-14,218		

Table S4. The manual function annotation of 11 genes within the melanin biosynthetic gene cluster in *A. pullulans* NRRL 62031 through Blastp alignment.

Gene domain	Gene description	Identity	E value	Accession number
a	Hypothetical protein hypothetical protein QM012_007263 [*Aureobasidium pullulans*]	100%	0.0	KAK6005621.1
b	Hypothetical protein QM012_007262 [*Aureobasidium pullulans*]	100%	2e-93	KAK6005620.1
c	S-adenosyl-L-methionine-dependent methyltransferase [*Aureobasidium melanogenum*]	84.26%	0.0	KAH0093470.1
d	JAB1/MPN domain-containing protein [*Aureobasidium pullulans*]	91.36%	0.0	KAG9636444.1
e	Hypothetical protein QM012_007259 [*Aureobasidium pullulans*]	94.14%	0.0	KAK6005617.1
f	Hypothetical protein QM012_007258 [*Aureobasidium pullulans*]	100%	0.0	KAK6005616.1
g	Prefoldin, subunit 3 [*Aureobasidium melanogenum*]	92.31%	3e-125	KAG9546454.1
h	Polyketide synthase [*Aureobasidium melanogenum*]	96.68%	0.0	ALB35145.1
i	Transcription factor Cmr1 [*Aureobasidium melanogenum* CBS 110374]	97.74%	0.0	XP_040884520.1
j	ESC (Elsinoë fawcetti) reductase [*Aureobasidium melanogenum* CBS 110374]	99.62%	0.0	XP_040884521.1
	Putative tetrahydroxynaphthalene reductase [*Fulvia fulva*]	90.46%	7e-177	XP_047768494.1
k	Disulfide isomerase PDI1 protein [*Aureobasidium melanogenum*]	99.74%	0.0	KAG9632850.1

Table S5. The functional annotation of putative key enzyme-encoding genes responsible for the biosynthesis and regulation of melanin in *A. pullulans* NRRL 62031.

Gene_ ID	Nr_ID	Nr_Description	Identity (%) / E-value	Kegg_EC	Kegg_ Definition	Identity (%)/ E-value
**Scytalone dehydratase (SCD1)**						
g7053	KEQ60876.1	scytalone dehydratase [*Aureobasidium melanogenum* CBS 110374]	98.9/1.1E-100	4.2.1.94	scytalone dehydratase	77.8 / 2E-79
**Tetrahydroxynaphthalene reductase (T4HR1)**						
g2264	KEQ67498.1	ESC reductase [*Aureobasidium melanogenum* CBS 110374]	99.6 / 9.9E-146	1.1.1.252	Tetrahydroxy-naphthalene reductase	89.3 / 1.6E-133
g5807	KEQ79181.1	hypothetical protein M438DRAFT_339949 [*Aureobasidium pullulans* EXF-150]	70.9 / 1.2E-199	1.1.1.252	Tetrahydroxy-naphthalene reductase	67.1 / 7.7E-83
**Tyrosinase (TYR)**						
g941	KEQ58885.1	tyrosinase central domain-containing protein [*Aureobasidium melanogenum* CBS 110374]	87.7 / 3.7E-205	1.14.18.1	tyrosinase	55.1 / 1.3E-123
g1154	XP_013348277.1	hypothetical protein AUEXF2481DRAFT_61752 [*Aureobasidium subglaciale* EXF-2481]	79.7 / 0	1.14.18.1	tyrosinase	55.5 / 6.5E-125
g3541	KEQ62328.1	FAD / NAD(P)-binding domain-containing protein [*Aureobasidium melanogenum* CBS 110374]	89.8 / 1.5E-195	1.14.18.1	tyrosinase	46.0 / 1E-91
g5277	KEQ60213.1	Di-copper centre-containing protein [*Aureobasidium melanogenum* CBS 110374]	89.8 / 1.5E-167	1.14.18.1	tyrosinase	38.1 / 9.90E-16
g7018	KEQ58304.1	Di-copper centre-containing protein [*Aureobasidium melanogenum* CBS 110374]	92.1 / 4.9E-224	1.14.18.1	tyrosinase	59.8 / 1.1E-131
g8919	KEQ57877.1	hypothetical protein M437DRAFT_89107 [*Aureobasidium melanogenum* CBS 110374]	100 / 4.1E-133	1.14.18.1	tyrosinase	51.9 / 1.50E-14
**Tyrosine aminotransferase (TAT)**						
g8054	KEQ63246.1	PLP-dependent transferase [*Aureobasidium melanogenum* CBS 110374]	97.2 / 4.7E-224	2.6.1.5	tyrosine aminotransfer ase	25.6 / 8E-23
**4-hydroxyphenylpyruvate dioxygenase (HppD)**						
g783	XP_013348491.1	hypothetical protein AUEXF2481DRAFT_203197 [*Aureobasidium subglaciale* EXF-2481]	91.4 / 3.7E-216	1.13.11.27	4-hydroxy-phenyl-pyruvate dioxygenase	72.3 / 9.5E-173
g4074	XP_013428521.1	xylose isomerase-like protein [*Aureobasidium namibiae* CBS 147.97]	81.6 / 9.5E-165	1.13.11.27	4-hydroxy-phenyl-pyruvate dioxygenase	60.5 / 1.4E-121
g4754	KEQ63052.1	putative 3-dehydroshikimate dehydratase [*Aureobasidium melanogenum* CBS 110374]	96 / 8E-196	1.13.11.27	4-hydroxy-phenyl-pyruvate dioxygenase	36.9 / 8.9E-47

g6112	KEQ62272.1	3-dehydroshikimate dehydratase [*Aureobasidium melanogenum* CBS 110374]	92.9 / 1.1E-192	1.13.11.27	4-hydroxy-phenyl-pyruvate dioxygenase	58.8 / 4.6E-115
**Transcription factor Cmr1**						
g2263	XP_013431775.1	transcription factor Cmr1 [*Aureobasidium namibiae* CBS 147.97]	94.8 / 0	--	KRAB domain-containing zinc finger protein	30.8 / 1.80E-07

Table S6. The functional annotation of putative transcription factor-encoding genes against the ATFDB (*Aureobasidium* transcription factor database) in *A. pullulans* NRRL 62031

Gene_ ID	Transcription factor	Regulation	Domain	Accession no.	Identity (%) / E-value
g640	Swi4/6 (Specific)	CWI (cell wall integrity) Pathway	KilA-N domain	KEQ58325.1	98.6 / 0
g2263	Cmr1/Pig1 (Specific)	Regulation of secondary metabolites (SM)	zf-H2C2_2 (Zinc-finger double domain); Zn(2)-Cys(6) binuclear cluster domain	XP_013431775.1	94.83 / 0
g7025	Msn2 (Global)	Regulation of nitrogen metabolism	zf-H2C2_2 (Zinc-finger double domain)	XP_013341874.1	81.65 / 0
g8040	PacC/Rim101 (Specific)	Regulation of acidic and alkaline genes by environmental pH	zf-H2C2_2 (Zinc-finger double domain)	KEQ83946.1	88.39 / 0
g161	Crz1p/Tcn1p/Hal8 (Global)	Calcium signaling pathway	zf-H2C2_2 (Zinc-finger double domain)	KEQ62890.1	97.2 / 0

Table S7. The functional annotation of putative key enzyme-encoding genes responsible for the biosynthesis and regulation of pullulan in *A. pullulans* NRRL 62031.

Gene_ ID	Nr_ID	Nr_Description	Identity (%) / E-value	Kegg_ EC	Kegg_Definition	Identity (%) / E-value
**α-phosphoglucose mutase (Pgm)**						
g852	KEQ59353.1	phosphoglucomutase-2 [*Aureobasidium melanogenum* CBS 110374]	90.1 / 1E-307	5.4.2.2	Phosphogluco-mutase	62.4 / 2.3E-204
g7458	XP_007780174.1	phosphoglucomutase [*Coniosporium apollinis* CBS 100218]	89.5 / 4.1E-295	5.4.2.2	Phosphogluco-mutase	87.5 / 2.1E-295
**UDP-glucose pyrophosphorylase (Ugp)**						
g4829	XP_023890373.1	probable UTP-glucose-1-phosphate uridylyltransferase [*Quercus suber*]	91.2 / 2.1E-269	2.7.7.9	UTP-glucose-1-phosphate uridylyltransferase	89.8 / 6.4E-267
**UDP-glucose:glycoprotein glucosyltransferase**						
g2658	AQQ13387.1	UDP-glucosyltransferase [*Aureobasidium melanogenum*]	93.7 / 0	2.4.1.-	UDP-glucose: glycoprotein glucosyl-transferase	59 / 0
**Sterol glucosyltransferase (SGT1)**						
g2104	KEQ62498.1	sterol glucosyltransferase [*Aureobasidium melanogenum* CBS 110374]	85.8 / 0	6.1.1.5	isoleucyl-tRNA synthetase	47.1 / 4.6E-153
**Ceramide glucosyltransferase (Gcs1)**						

g4364	KEQ61150.1	hypothetical protein M437DRAFT_52539 [*Aureobasidium melanogenum* CBS 110374]	95.5 / 3.3E-305	2.4.1.80	ceramide glucosyl-transferase	60.5 / 4.6E-153
**Glycogenin**						
g840	KEQ81970.1	nucleotide-diphospho-sugar transferase [*Aureobasidium pullulans* EXF-150]	68.1 / 6.3E-141	2.4.1.186	glycogenin	32.8 / 2.7E-20
g7961	KEQ83942.1	nucleotide-diphospho-sugar transferase [*Aureobasidium pullulans* EXF-150]	79.9 / 0	2.4.1.186	glycogenin	53.2/1.5E-185
**Glycogen synthase (Gys)**						
g8879	GAM87952.1	hypothetical protein ANO11243_059800 [fungal sp. No.11243]	90.8 / 0	2.4.1.11	glycogen synthase	88.6/0
**α-amylase (Amy)**						
g1316	AEH03024.1	α-amylase [*Aureobasidium pullulans*]	95.2 / 0	3.2.1.1	α-amylase	65.9/2.9E-234
g3298	KEQ65306.1	α-amylase [*Aureobasidium melanogenum* CBS 110374]	90.0 / 3.4E-264	3.2.1.1	α-amylase	58.4/5.7E-172
g3502	KEQ65480.1	α-amylase [*Aureobasidium melanogenum* CBS 110374]	93.9 / 7.5E-280	3.2.1.1	α-amylase	62.6/5E-184
g6635	KEQ67332.1	glucan 1,4-α-maltohexaosidase precursor [*Aureobasidium melanogenum* CBS 110374]	85.6 / 7E-295	3.2.1.1	α-amylase	58.6/6.8E-166
g8572	KEQ61316.1	Putative α-amylase [*Aureobasidium melanogenum* CBS 110374]	96.7 / 0	3.2.1.1	α-amylase	70.2/7.3E-218
**α-glucan synthase (GS)**						
g5175	XP_013428544.1	Putative α-1,3 glucan synthase [*Aureobasidium namibiae* CBS 147.97]	89.4 / 0	2.4.1.183	α-1,3-glucan synthase	58.2/0
g6809	AYG85498.1	α-glucan synthase, partial [*Aureobasidium melanogenum*]	96 / 0	2.4.1.183	α-1,3-glucan synthase	65.7/0
g7000	AYG85496.1	α-glucan synthase [*Aureobasidium melanogenum*]	90.6 / 0	2.4.1.183	α-1,3-glucan synthase	48.2/0
**Transcription factor CreA**						
g274	AIZ77451.1	DNA-binding protein creA [*Aureobasidium pullulans*]	99.8 / 2E-274	--	zinc-finger protein CreA/MIG	67.7/1.3E-165
**Transcription factor AreA**						
g7383	AWD76385.1	nitrogen regulatory protein AreA [*Aureobasidium melanogenum*]	96.3 / 0		GATA-binding protein, other eukaryote	46.7/3E-208
**Transcription factor AreB**						
g4625	AWD76386.1	nitrogen regulatory protein AreB [*Aureobasidium melanogenum*]	97.4 / 3.2E-167		GATA-binding protein, other eukaryote	57.7/1.2E-77

Table S8. The functional annotation of putative key enzyme-encoding genes responsible for the biosynthesis and regulation of polymalate in *A. pullulans* NRRL 62031.

Gene ID	Nr_ID	Nr_Description	Ident. (%) / E-value	Kegg_ EC	Kegg_Definition	Ident. (%) / E-value
**Pyruvate carboxylase (PYC)**						
g667	KEQ58358.1	hypothetical protein M437DRAFT_79067 [*Aureobasidium melanogenum* CBS 110374]	84.8 / 5.7E-271	6.4.1.1	pyruvate carboxylase	39.5 / 1.8E-84
g6960	OQO07440.1	Pyruvate carboxylase [*Rachicladosporium antarcticum*]	87.5 / 0	6.4.1.1	pyruvate carboxylase	86.2 / 0
**Malate dehydrogenase (MDH)**						
g2315	KEQ67181.1	hypothetical protein M437DRAFT_38006 [*Aureobasidium melanogenum* CBS 110374]	88.6 / 0	1.1.1.38	malate dehydrogenase (oxaloacetate-de-carboxylating)	67.7 / 5E-239
g2655	KEQ83171.1	hypothetical protein M438DRAFT_346806 [*Aureobasidium pullulans* EXF-150]	96.5 / 0	1.1.1.40	malate dehydrogenase (oxaloacetate-de-carboxylating)($NADP^+$)	79.3 / 3.9E-257
g4110	KEQ58185.1	malate dehydrogenase [*Aureobasidium melanogenum* CBS 110374]	97.6 / 7.1E-181	1.1.1.37	malate dehydrogenase	82.3 / 1.6E-154
g5765	ANE21355.1	malate dehydrogenase [*Aureobasidium* sp. P25]	93.3 / 2.4E-173	1.1.1.37	malate dehydrogenase	80.0 / 1.4E-150
**Malate synthase (MSE)**						
g7480	OCL10598.1	malate synthase [*Glonium stellatum*]	86 / 2E-278	2.3.3.9	malate synthase	84.7 / 1E-275
**Fumarase (FUM)**						
g6753	KEQ81564.1	tubulin nucleotide-binding domain-like protein [*Aureobasidium pullulans* EXF-150]	91.3 / 9E-284	4.2.1.2	fumarate hydratase, class II	31.7 / 2E-42
g7477	GAM89660.1	hypothetical protein ANO11243_076990 [fungal sp. No.11243]	85.9 / 3.4E-243	4.2.1.2	fumarate hydratase, class II	84.2 / 2.6E-236
**Phosphopantetheinyl transferase (PPTase)**						
g2273	AST22499.1	phosphopantetheinyl transferase [*Aureobasidium melanogenum*]	94.1 / 5.8E-183	2.7.8.-	4'-phosphopante-theinyl transferase	59.1 / 6.8E-116
**Transcription factor NsdD**						
g233	AXS67923.1	putative transcription factor NsdD [*Aureobasidium melanogenum*]	95.2 / 1E-268		structural maintenance of chromosome 3 (chondroitin sulfate proteoglycan 6)	63.9 / 6.00E-07

Table S9. The functional annotation of putative key enzyme-encoding genes responsible for the biosynthesis of polyol lipids in *A. pullulans* NRRL 62031.

Gene_ID	Nr_ID	Nr_Description	Ident. (%) / E-value	Kegg_ EC	Kegg_Definition	Ident. (%) / E-value
**Mannitol-1-phosphate 5-dehydrogenase (MPDH)**						
g284	OBW69787.1	Cat eye syndrome critical region protein 5 [*Aureobasidium pullulans*]	93.0 / 3.7E-202	1.1.1.17	mannitol-1-phosphate 5-dehydrogenase	72.3 / 9E-157

**Mannitol dehydrogenase (MtDH)**						
g3029	AST36438.1	putative mannitol dehydrogenase [*Aureobasidium melanogenum*]	99.6 / 3.7E-148			76.3 / 7E-116
**Arabitol dehydrogenase (ArDH)**						
g5185	AYC07633.1	putative arabitol dehydrogenase [*Aureobasidium melanogenum*]	99.7 / 6.5E-212	1.1.1.14	L-iditol 2-dehydrogenase	79.5 / 2E-174
**Highly reducing polyketide synthase (HR-PKS)**						
g2599	AND82609.1	polyketide synthase [*Aureobasidium melanogenum*]	94.6 / 0		emericellamide biosynthesis, highly reducing iterative type I polyketide synthase	36.8 / 1.4E-233
**Esterase (Est1)**						
g2600	AYC07631.1	esterase [*Aureobasidium melanogenum*]	91.8 / 1.2E-141			
**Phosphopantetheinyl transferase (PPTase)**						
g2273	AST22499.1	phosphopantetheinyl transferase [*Aureobasidium melanogenum*]	94.1 / 5.8E-183	2.7.8.-	4'-phosphopantetheinyl transferase	59.1 / 6.8E-116

Table S10. The function annotation of putative key enzyme-encoding genes responsible for the biosynthesis of fructooligosaccharides, gluconic acid, and β-glucan in *A. pullulans* NRRL 62031.

Gene ID	Nr_ID	Nr_Description	Ident. (%) / E-value	Kegg_ EC	Kegg_Definition	Ident. (%) / E-value
**β-fructofuranosidase**						
g1443	ARG41450.1	β-fructofuranosidase [*Aureobasidium melanogenum*]	95.1 / 0	3.2.1.26	β-fructofuranosidase	41.7 / 1.2E-119
g3017	KIV84871.1	hypothetical protein PV11_00623 [*Exophiala sideris*]	54.9 / 8.9E-162	3.2.1.26	β-fructofuranosidase	54.1 / 6.2E-140
g7938	ARG41451.1	β-fructofuranosidase [*Aureobasidium melanogenum*]	95.2 / 0	3.2.1.26	β-fructofuranosidase	59.6 / 1.7E-188
g7939	KEQ67434.1	glycosyl hydrolase-like proteins family 32 superfamily [*Aureobasidium melanogenum* CBS 110374]	95.7 / 0	3.2.1.26	β-fructofuranosidase	62.3 / 1.4E-231
**Glucose oxidase**						
g1252	KEQ67892.1	putative glucose oxidase [*Aureobasidium melanogenum* CBS 110374]	82.5 / 2.7E-289	1.1.99.1	choline dehydrogenase	35.4 / 1.4E-92
g1296	ASN67018.1	glucose oxidase [*Aureobasidium pullulans*]	91.7 / 0	1.1.99.1	choline dehydrogenase	40.5 / 3.3E-118
**1,3-β-glucan synthase**						
g2024	XP_013342181.1	glycosyltransferase family 48 protein [*Aureobasidium subglaciale* EXF-2481]	95.5 / 0	2.4.1.34	1,3-β-glucan synthase	83.4 / 0

References

Černoša, A., Sun, X., Gostinčar, C., Fang, C., Gunde-Cimerman, N., and Song, Z. (2021). Virulence Traits and Population Genomics of the Black Yeast *Aureobasidium melanogenum*. Journal of fungi (Basel, Switzerland) 7.

Gostinčar, C., Ohm, R.A., Kogej, T., Sonjak, S., Turk, M., Zajc, J., Zalar, P., Grube, M., Sun, H., Han, J., et al. (2014). Genome sequencing of four *Aureobasidium pullulans* varieties: biotechnological potential, stress tolerance, and description of new species. BMC genomics 15, 549.

Gostinčar, C., Turk, M., Zajc, J., and Gunde-Cimerman, N. (2019). Fifty *Aureobasidium pullulans* genomes reveal a recombining polyextremotolerant generalist. Environmental Microbiology 21, 3638-3652.

Onetto, C.A., Schmidt, S.A., Roach, M.J., and Borneman, A.R. (2020). Comparative genome analysis proposes three new *Aureobasidium* species isolated from grape juice. FEMS yeast research 20.
